# Dysregulation of a specific immune-related network of genes biologically defines a subset of schizophrenia

**DOI:** 10.1038/s41398-019-0486-6

**Published:** 2019-05-31

**Authors:** Svenja V. Trossbach, Laura Hecher, David Schafflick, René Deenen, Ovidiu Popa, Tobias Lautwein, Sarah Tschirner, Karl Köhrer, Karin Fehsel, Irina Papazova, Berend Malchow, Alkomiet Hasan, Georg Winterer, Andrea Schmitt, Gerd Meyer zu Hörste, Peter Falkai, Carsten Korth

**Affiliations:** 1 Department Neuropathology, Medical Faculty, Heinrich Heine University Düsseldorf, Düsseldorf, Germany; 2 Department of Neurology, Westfälische Wilhelms-University, Münster, Germany; 3 Biological and Medical Research Center (BMFZ), Genomics and Transcriptomics Laboratory (GTL), Medical Faculty, Heinrich Heine University Düsseldorf, Düsseldorf, Germany; 4 Institute of Quantitative and Theoretical Biology, Heinrich Heine University Düsseldorf, Düsseldorf, Germany; 5 Department of Psychiatry and Psychotherapy, Medical Faculty, Heinrich Heine University Düsseldorf, Düsseldorf, Germany; 6 Department of Psychiatry and Psychotherapy, Ludwig-Maximilians-University, Munich, Germany; 7 Laboratory of Neuroscience (LIM27), Institute of Psychiatry, University of Sao Paulo, Rua Dr. Ovidio Pires de Campos 785, 05453-010 São Paulo - SP – Brazil, Sao Paulo, Brazil; 8 Present Address: University Children’s Hospital, University Medical Center Hamburg-Eppendorf, Hamburg, Germany; 9 Present Address: Thermo Fisher Scientific Life Technologies GmbH, Darmstadt, Germany; 10 Present Address: Biological and Medical Research Center (BMFZ), Genomics and Transcriptomics Laboratory (GTL), Medical Faculty, Heinrich Heine University Düsseldorf, Düsseldorf, Germany; 11 Present Address: Clinical Neuroscience Research Group, Experimental and Clinical Research Center (ECRC), Dept. of Anesthesiology and Operative Intensive Care Medicine (CCM, CVK), Charité – Universitätsmedizin Berlin, Corporate Member of Freie Universität Berlin, Humboldt-Universität zu Berlin, and Berlin Institute of Health (BIH), Berlin, Germany

**Keywords:** Diagnostic markers, Schizophrenia

## Abstract

Currently, the clinical diagnosis of schizophrenia relies solely on self-reporting and clinical interview, and likely comprises heterogeneous biological subsets. Such subsets may be defined by an underlying biology leading to solid biomarkers. A transgenic rat model modestly overexpressing the full-length, non-mutant Disrupted-in-Schizophrenia 1 (DISC1) protein (tgDISC1 rat) was generated that defines such a subset, inspired by our previous identification of insoluble DISC1 protein in *post mortem* brains from patients with chronic mental illness. Besides specific phenotypes such as DISC1 protein pathology, abnormal dopamine homeostasis, and changes in neuroanatomy and behavior, this animal model also shows subtle disturbances in overarching signaling pathways relevant for schizophrenia. In a reverse-translational approach, assuming that both the animal model and a patient subset share common disturbed signaling pathways, we identified differentially expressed transcripts from peripheral blood mononuclear cells of tgDISC1 rats that revealed an interconnected set of dysregulated genes, led by decreased expression of regulator of G-protein signaling 1 (RGS1), chemokine (C–C) ligand 4 (CCL4), and other immune-related transcripts enriched in T-cell and macrophage signaling and converging in one module after weighted gene correlation network analysis. Testing expression of this gene network in two independent cohorts of patients with schizophrenia versus healthy controls (*n* = 16/50 and *n* = 54/45) demonstrated similar expression changes. The two top markers RGS1 and CCL4 defined a subset of 27% of patients with 97% specificity. Thus, analogous aberrant signaling pathways can be identified by a blood test in an animal model and a corresponding schizophrenia patient subset, suggesting that in this animal model tailored pharmacotherapies for this patient subset could be achieved.

## Introduction

Despite decades of scientific efforts, schizophrenia or other chronic mental illnesses (CMI) in clinical psychiatry lack an objective biological diagnostic test. Diagnosis in clinical psychiatry still relies on the subjective clinical interview and, paradoxically, biology-based diagnostics is used only to exclude conditions, such as trauma, drug intoxication, or “neurological” causes.

The predicament for a biological definition of mental illness results from the fact that all efforts for the discovery of biological underpinnings start with the clinical diagnosis that pools patients from heterogeneous biological subsets leading to a dilution of possible size effects of specific markers for a disease subset. This problem cannot be solved by including larger patient numbers because ratios of biological subsets within the clinical “umbrella” diagnosis will remain the same.

For example, schizophrenia is, in most cases, a chronic disease clinically diagnosed with positive (hallucinations, delusions, thought disorder), negative (flattened affect, lack of drive), and cognitive symptoms (working memory and attention deficits). The diversity of underlying biology in schizophrenia patients is already mirrored by variations in chronic impairment and biological time courses^1^. As repeatedly demonstrated on the genetic level, there is strong overlap of schizophrenia with recurrent affective disorders^2–4^ arguing against the value of a purely clinical diagnosis for investigating underlying biology. Attempts to identify common genetic variants increasing the risk for obtaining the clinical diagnosis schizophrenia led to the discovery of over 108 associated loci with low logarithm of odds (LOD) scores of all genes of below 18, except MHCII with a LOD score of 30 (ref. ^5^). These relatively small effect sizes could in part be due to the “dilution of subsets” argument outlined above.

Ever since the discovery of the genetic component of schizophrenia, it has also been recognized that a large portion of the disease risk is actually non-genetic^6^. We previously followed up the idea of subtle protein misassembly or aggregation as one feature of aberrant protein homeostasis in chronic mental illnesses(CMI)^7^. Since the same proteins are detected in insoluble deposits in the majority of sporadic cases as in the rare familial cases of neurodegenerative diseases^8^, we reasoned that the products of genes with rare mutations in familial schizophrenia would be good candidates for protein misassembly^9^. In the neurodegenerative diseases, most mutations leading to aggregating proteins are rare mutations and, for example, in Alzheimer’s disease, do not display common variants^10^. We chose the *Disrupted-in-Schizophrenia 1* (*DISC1*) gene that is mutated both in a Scottish family^11^ and in an American family^12^ and associates with schizophrenia as well as affective disorders thus crossing current diagnostic boundaries. Its genetic linkage to mental illnesses has also been reported from Finnish families^13,14^. When we investigated a collection of *post mortem* brains from patients with schizophrenia, major depression, bipolar disorder, and healthy controls for the presence of insoluble DISC1, we identified biochemically insoluble DISC1 in ~15% of cases with different clinical diagnoses^9^, but not healthy individuals or controls with various neurodegenerative diseases^15^. DISC1 overexpression in animals or cells leads to DISC1 protein misassembly or aggregation independent of mutations^9,15^,^16^, and no subsequent regulation of endogenous DISC1 protein levels has been observed^16^. When modeling the effects of misassembled, full-length, non-mutant DISC1 protein on brain functions by modestly overexpressing it as a transgene in a rat (tgDISC1 rat, with ~11-fold overexpression compared with endogenous rat Disc1)^16^, we observed a series of phenotypes in independent lines with high face validity to major mental disorders^16–19^. Behavioral deficits were compatible with dysregulation of dopamine homeostasis^16,20^, a major factor relevant for behavioral control, including in schizophrenia. Neurochemical and neuroanatomical changes confirmed the role of aberrant neurodevelopment of the dopamine system^21^, as well as attention and long-term memory deficits^19,20^, two features highlighted by the Measurement and Treatment Research to Improve Cognition in Schizophrenia (MATRICS) Initiative for translating cognitive features of schizophrenia to animal models^22^.

**Fig. 1:**
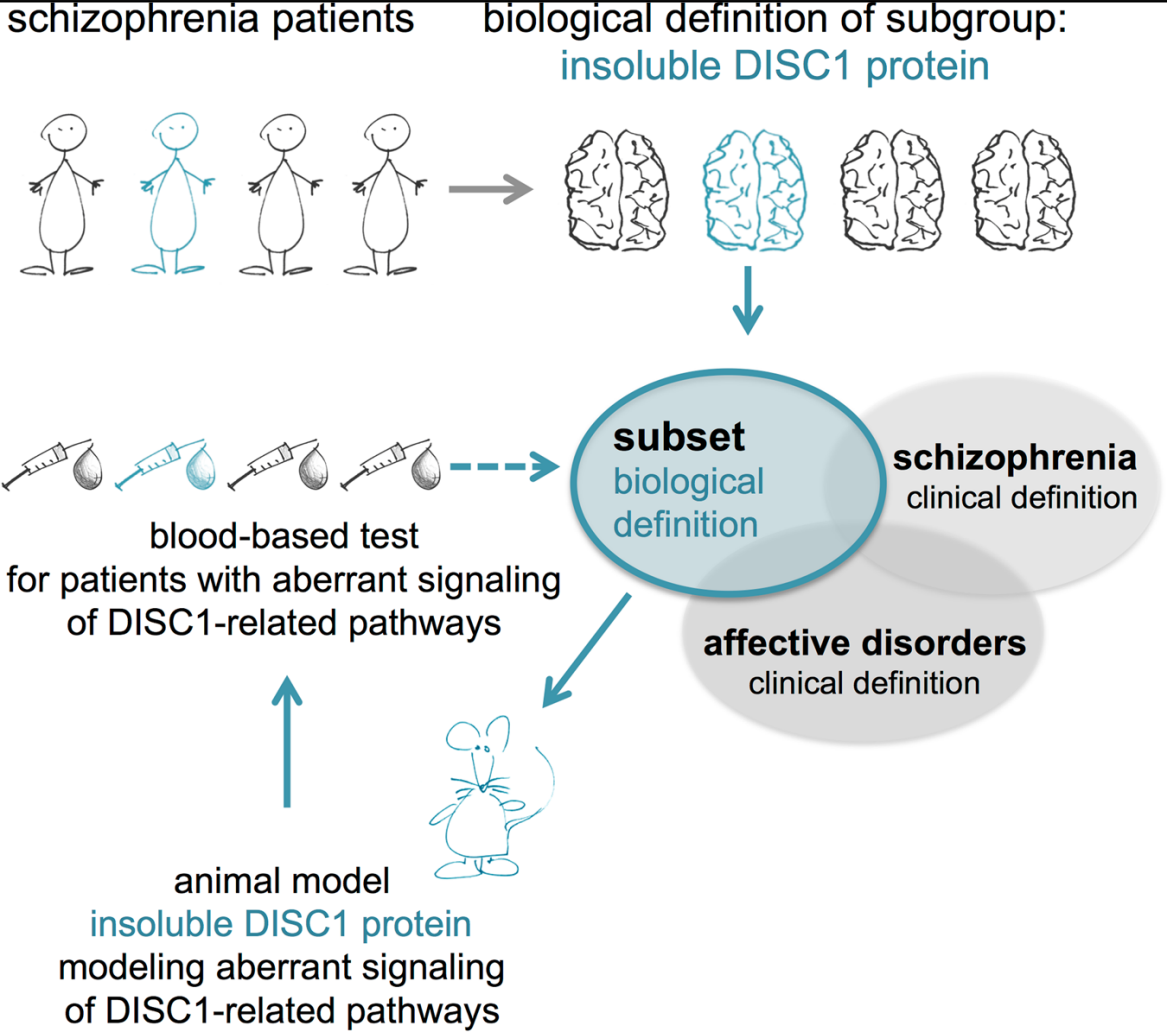
The reverse translation approach to biomarker discovery. The heterogeneous group of mental illness patients lacks a clear biology-based clinical categorization which impedes attempts for the discovery of objective biomarkers so far. By defining biological subgroups based on human neuropathobiochemistry, in our case misassembled DISC1 protein in *post mortem* brain^9^, we were able to design a transgenic rat model for this specific subset^16^ that demonstrated aberrant signaling networks of key pathways relevant for schizophrenia^27^. Utilizing this animal model as a prototype for subtly deranged signaling networks essential for behavioral control, peripheral markers were identified in this animal model which, in a so-called “reverse translational approach”, were translated back into schizophrenia patient cohorts, coming full circle for defining subsets of mental illness patients

Accumulated research from the last decade has shown that DISC1 is a scaffold-like protein that is participating in several mental illness-related signaling pathways and likely acts as a convergence point in these^7,23–26^. Therefore, a subtle dysregulation of DISC1 protein functionality as a result of its misassembly, resulting in both loss and gain-of-function phenotypes^9,15^, likely impacts several signaling pathways relevant for CMI^27^. Since the tgDISC1 rat demonstrates a phenotype for non-mutant DISC1 protein posttranslational alterations, i.e., protein misassembly, it is a more valid model for the majority of sporadic cases of CMI than a model with a DISC1 mutation or ablation and therefore represents a valid model for a subset of patients with sporadic schizophrenia. We hypothesized that given the central function of the DISC1 protein in CMI-relevant signaling pathways and their disruption in the tgDISC1 rat, we could identify biological markers in peripheral tissues of this animal model to serve as starting points for the discovery of similar markers in patients, thereby revealing a shared pathophysiology. In fact, we here successfully use this “reverse translation” approach to translate biological markers identified in an animal model to patients (see Fig. 1). Applying this novel approach for mental illnesses, we here describe a dysregulated network of immune-related genes, able to biologically classify a subset of 27% of schizophrenia patients with 97% specificity, and representing specific clinical characteristics.

## Materials and methods

### Subjects and classifications

The study was carried out following the rules of the Declaration of Helsinki of 1975, revised in 2008. All participants were fully informed about the procedures and gave written informed consent.

#### Group I–HHU

Control subjects and patients diagnosed with schizophrenia of group I were part of a clinical study as described earlier^28,29^. Demographic information regarding the cohort can be found in Supplementary Table 1, and further information is available in Warbrick et al.^28^ and Trossbach et al.^29^. The study protocol and its amendments were approved by the local ethics committee (Medical Faculty of the Heinrich Heine University Düsseldorf: EudraCT-Number: 2006-006329-19).

#### Group II - LMU

##### Sample

Patients were recruited at the Department for Psychiatry and Psychotherapy, Clinic of the University of Munich. The study protocol and its amendments were approved by the local ethics committee (Medical Faculty of the LMU Munich: Code 17–13; Date of Approval: 25th of February 2013 and 25th of March 2014).

##### Procedure

Using the Verbal Learning and Memory Test (VLMT)^30^, the Digit-Span-Test (DST, subtest of the Hamburg–Wechsler Intelligence Test)^31^, Digit Symbol Substitution Test (DSST, subtest of the Hamburg–Wechsler Intelligence Test)^31^, and the Trial Making Test A and B (TMT-A, TMT-B)^32^, we assessed verbal episodic memory, working memory, attention, and cognitive flexibility in Group II. The test results were standardized (z-values) and averaged to a memory, an attention, and a main cognition score serving as outcome variables (for details, see Supplementary Tables 2, 3). In addition, we assessed symptoms severity with the Positive and Negative Syndrome Scale (PANSS)^33^, medication, medical history, and demographics (Supplementary Table 1).

##### Statistical analysis (Group II –LMU)

We conducted all statistical analysis at significance level of α = 0.05 using SPSS 24 (IBM, Ehningen, Germany) for Windows. First, we compared cognitive performance between patients with and without marker combination (for explanation, see the Results section), using univariate ANOVAs. In case of violation of the assumption of homogeneity of variances, we applied Welch’s ANOVA. Then, we tested if and how gene expressions of RGS1, CCL4, DISC1, as well as the RGS1 + CCL4 combination are associated with cognitive functioning with Kendall’s tau correlation analysis. Demographic and clinical differences between both patient groups were carried out using ANOVAs. The results are presented without correction for multiple testing.

### Animals

Animal experiments were executed in conformity with the German Animal Protection Law and were authorized by local authorities (LANUV NRW, Recklinghausen, Germany). Blood extraction, splenocyte extraction, and preparation of PBMCs were performed with transgenic Sprague Dawley rats overexpressing full-length, non-mutant DISC1 (tgDISC1 rat, TG, see ref. ^16^ for full description) and non-transgenic littermates (LM). All rats were males and 8–9 months old. TgDISC1 and control rats were bred at the ZETT, Heinrich Heine University Düsseldorf, Germany. Animals were housed three animals per cage under standard laboratory conditions with lights on from 7 a.m. to 7 p.m. and with water and food provided *ad libitum*.

### Preparation of rat peripheral blood mononuclear cells (PBMCs) from blood

Anaesthetized rats underwent a heart puncture to harvest a minimum of 8 mL of blood with 10 mM EDTA as an anti-coagulant. Rat PBMCs were prepared with Ficoll-Paque Premium 1.084 solution (GE Healthcare, Little Chalfont, UK) according to the manufacturer’s instructions. Preparation of human PBMCs was performed with Ficoll-Paque Plus as described earlier^29^. All lymphocyte samples were snap-frozen in liquid nitrogen and stored at −80 °C until further processing.

### Preparation of rat splenocytes

The spleen was dissected out of rats (*n* *=* 5 TG and LM) and forced through a 70 µm cell strainer to get a single-cell suspension. The cell strainer was washed afterwards twice with PBS + 2% FCS.

### Antibody staining for flow cytometry

Rat blood and splenocytes (*n* = 5 each for TG and LM) were stained for 30 min at 4 °C with antibodies according to the manufacturer’s recommendations. Following antibodies were used: anti-rat CD3, FITC, clone 1F4, BioLegend; anti-rat CD4, PE-Cy7, clone W3/25, BioLegend; anti-rat CD8, Pe, clone G28, BioLegend; anti-rat CD45, BV510, clone OX-1, BD Biosciences; anti-rat CD45RA, PerCP-Cy5.5, clone OX-33, BioLegend; anti-rat CD161a, BV421, clone 10/78, BD Biosciences. After staining, cells were washed twice and erythrocytes were lysed using VersaLyseTM Lysing Solution (Beckman Coulter, Krefeld, Germany) according to the manufacturer’s recommendations. Afterward, cells were acquired with a GalliosTM (Beckman Coulter) flow cytometer and analyzed with FlowJo analysis software v10.4.1 (Tree Star, Inc., Ashland, USA).

### Generation of single-cell libraries and sequencing

Human PBMCs intended for single-cell sequencing were isolated using Lymphoprep (STEMCELL Technologies, Vancouver, Canada) according to the manufacturer’s instructions from a single healthy donor. In total, 150,000 single cells were processed with the Drop-Seq technique as previously described^34^ to capture about 3,500 cells. Library Preparation was carried out with the Nextera XT Kit (Illumina, San Diego, USA) according to the manufacturer’s instructions. Sequencing was performed on a local Illumina Nextseq 500 using the High-Out 75 cycle kit with a 20–8–0–63 read setup, aiming at 40,000–50,000 reads/cells.

### Single-cell RNA-sequencing data analysis

Raw sequencing data were preprocessed by the Drop-Seq tools v1.12 pipeline. A single cell gene expression data set derived from one healthy donor for 8,000 PBMCs was downloaded from ×10 Genomics (support.10xgenomics.com). Subsequent analysis was carried out with the R-package Seurat v2.1^35^, using R v3.4.3 and RStudio v1.1.383. In a first step, low-quality cells and cell duplets were removed from the data set. The data were then normalized and variable genes were identified by calculating the average expression and dispersion for each gene. Linear dimensional reduction was done using Principal Component Analysis (PCA) using the identified variable genes as input. Statistically significant Principal Components (PCs) were identified by a combination of a JackStraw significance test and plotting standard deviations of the PCs. Cell clustering was based on a graph-based clustering approach, employing K-nearest neighbor (KNN), and then visualized by t-distributed stochastic neighbor embedding (t-SNE). Differentially expressed genes were identified by a tobit-censoring model for zero inflated data^35^.

### Preparation of RNA and cDNA

RNA of rat and human PBMCs was prepared utilizing the RNeasy Mini Kit according to manufacturer’s guidelines. Residual genomic DNA was digested on column by the RNase-free DNaseI Set (both Qiagen, Hilden, Germany). RNA from PBMCs collection was diluted to a concentration of 100 ng/µL and 1 µg was used as input for the production of cDNA with the RevertAid Minus H First Strand Synthesis Kit in a total of 20 µL utilizing the random hexamer primers provided by the kit (Thermo Fisher Scientific, Waltham, MA, USA). The resulting cDNA was diluted 1:50, 1:25, or 1:10 dependent on the PCR results as indicated in Supplementary Table 4, and 5 µL were used as template input. All analyses were performed blind to diagnosis.

### Gene expression profiling

The total RNA preparations were checked for RNA integrity by Agilent 2100 Bioanalyzer quality control. All samples in this study showed high-quality RNA integrity numbers (RIN >9). RNA was further analyzed by photometric Nanodrop measurement and quantified by fluorometric Qubit RNA assays (Life Technologies, Waltham, USA).

Synthesis of biotin-labeled cDNA was performed on ten replicates of each experimental group (DISC1 transgenic (TG) rats and littermate (LM) controls, respectively), according to the manufacturer's protocol (WT Plus Reagent Kit; Affymetrix Inc, Waltham, USA). Briefly, 100 ng of the total RNA were converted to cDNA. After amplification by in vitro transcription and second cycle synthesis, cDNA was fragmented and biotin labeled by terminal transferase. Finally, end-labeled cDNA was hybridized to Affymetrix Rat Gene 2.0 ST Gene Expression Microarrays for 16 h at 45 °C, stained by strepatavidin/phycoerythrin conjugate, and scanned as described in the manufacturer's protocol.

Three samples (2x TG, 1x LM) did not pass hybridization quality control, two additional samples (3x TG, 2x LM) had to be excluded from further analyses because of a non-transgene-related malformation diagnosed after termination, leading to a final *n* = 7 for LM and *n* = 5 for TG.

The data analyses on 12 Affymetrix CEL files were conducted with GeneSpring GX software (Vers. 12.5; Agilent Technologies, Santa Clara, USA). Probes within each probeset were summarized by GeneSprings’ ExonRMA16 algorithm after quantile normalization of probe-level signal intensities across all samples to reduce inter-array variability. Input data pre-processing was concluded by baseline transformation to the median of all samples.

To further improve signal-to-noise ratio, a given probeset had to be expressed above background (i.e. fluorescence signal of a probeset was detected within the 20^th^ and 100^th^ percentiles of the raw signal distribution of a given array) in all replicates in at least one of two, or both conditions to be subsequently analyzed in pairwise comparison.

Differential gene expression was statistically determined by moderated *T* test^36^. The significance threshold was set to *p* = 0.01. No correction for multiple testing was applied.

### Coexpression network WGCNA analysis

#### Gene coexpression network analysis

The normalized Affymetrix data from the differential gene-expression profiling was used to build a coexpression network using the “WGCNA” R package^37^. The data set contained 29,489 expression values from 12 samples. Samples encompass the five DISC1 transgenic (TG) rats and seven littermate (LM) controls. Following the steps described in Langfelder, P. and Horvath^37^, we first build an adjacency matrix *A*_*m,n*_ based on Pearson’s pairwise correlation between gene (*m* and *n*) raised to a power β (soft thresholding) *A*_*m,n*_ = |cor(*m,n*)|^β in order to emphasize high correlations at the expense of low correlations. The soft thresholding parameter (β = 6) was defined using the R function “pickSoftThreshold” from the WGCNA package. Using this approach, we construct an unsigned correlation network considering absolute correlation values. Therefore, we are able to identify highly co-expressed genes and test only for differences between TG and LM rats. In a next step, the adjacency data were used to build the topologic overlap matrix (TOM), which is a measure of connectivity of a particular gene as the sum of its adjacency with all other genes from the network. The corresponding dissimilarity, which is used as a proxy for distance between a pair of genes, was therefore defined as 1-TOM.

#### Identification of significant modules

To classify genes with similar expression pattern into gene modules, hierarchical clustering was performed using the TOM dissimilarity as distance and average linkage as clustering method. Distance between modules was inferred from the dissimilarity between module eigengenes (MEs), this is the first principal component of a given module. Modules having short distances to each other were merged together resulting in a total of 104 modules. In order to identify modules and genes with expression level related to clinical traits (TG or LM), Pearson’s correlation between module eigengenes (MEs), gene expression, and trait information was performed. Significance was calculated using the R function “corPvalueStudent” from the WGCNA package. Ten out of 104 modules have a *p*-value lower 0.05, and were considered significant. To be very strict in our analysis, we choose the module (hotpink4) with highest correlation (*ρ* *=* 0.7429) and lowest *p*-value (0.0056), respectively to the trait information for further evaluation.

#### Identification of hub genes

Hub genes are highly interconnected genes in a module, and can be considered to be functionally central. In our study, hub genes were defined by absolute values of Pearson’s correlation of module membership > 0.8 and trait information, gene trait significance and intra-module connectivity >0.5. Due to the fact that we used an unsigned network, in a second step the module members were separated into two subsets according to their correlation sign, which represents (i) the reduced expression level in TG compared with LM (negative correlation) and (ii) elevated level of expression in TG compared with LM (positive correlation). The top 10 hub genes for each subset sorted by mean correlation, intra-module connectivity, and module.

#### Stress centrality

The subset with reduced expression level in TG samples compared with LM was used for further centrality analysis. To find out which node in the module is most “important” or possesses high “control” to the network, we measured the stress centrality for each node using Cytoscape^38^ NetworkAnalyzer module. Stress centrality, introduced by Shimbel^39^ is based on enumeration of these shortest paths. The goal of defining this measure is to find the amount of “work” done by each node in the network. In a network representation, the nodes correspond to the genes and edges to the transformed Pearson’s correlation from the adjacency matrix with values >0.2. The node size is proportional to the importance (stress centrality) of the node in the network with Rgs1 having the highest number (424).

### Quantitative expression analysis

For the verification of differential expression, target primers were tested by PCR using the HotStarTaq (Qiagen, Hilden, Germany). Effective primers were used for quantitative real-time PCR (qPCR) with the StepOnePlus Real-Time PCR System (Applied Biosystems, Carlsbad, CA, USA) and the Platinum SYBR Green qPCR SuperMix-UDG (Invitrogen, Carlsbad, CA, USA) in MicroAMP Fast Optical 96-Well Reaction Plates (Applied Biosystems, Carlsbad, CA, USA). Depending on the target, 5% Factor Q solution (Qiagen, Hilden, Germany) was added to the mix. A detailed list of primers used and additional information, as well as the *n* for every single analysis can be found in Supplementary Table 4. QPCR conditions: 10 min at 95 °C, 40 cycles of 15 s at 95 °C, and 60 °C for 1 min. The resulting data were processed with the corresponding StepOne Software v2.3 (Thermo Fisher Scientific, Waltham, MA, USA). The expression of the respective target was normalized to the expression level of the housekeeping gene *Actin* (rat) or *ARF1* (human), as well as against a rat or human PBMC control cDNA per plate to minimize variances between runs.

### SMRI (Stanley Medical Research Institute) array collection of brain RNA

The array collection of purified RNA from cohorts of patients with schizophrenia, bipolar disorder, and healthy controls from the BA9 region was obtained from the Stanley Medical Research Institute (www.stanleyresearch.org). Area BA9 was used due to availability of the RNA samples and is a location of interest for schizophrenia or bipolar disorders^40,41^. A detailed list of sample demographics and tissue information can be found online (http://www.stanleyresearch.org/brain-research/array-collection/). A revised table, summarizing the patient and tissue information of cases shown, can be found in Supplementary Table 5. CDNA synthesis and qPCR was performed as described in the section “Quantitative expression analysis”, with the only difference that 5 µL of 1:5 diluted cDNA was used as input. The integrity of the RNA was tested using the Fragment Analyzer (Advanced Analytical Technologies, Thermo Fisher, USA). Only RNA with an integrity score (RNA Quality Number; RQN) >8 was used for further analysis. For normalization, the housekeeping gene ARF1 was used. After the blind analysis of the qPCR, all samples with a Ct value >29 for RGS1 were considered as showing no expression of RGS1 in the brain.

### Statistics

Statistical analyses were performed with the IBM SPSS Statistic program (IBM, Ehningen, Germany) or GraphPad Prism Version 6 (GraphPad Software Inc., San Diego, CA, USA). All data sets were tested for normal distribution, and appropriate parametric or non-parametric tests were chosen. Two datasets were tested by two-tailed Student’s *t* test or Mann–Whitney U test, respectively, if not stated otherwise. Correlations were analyzed by Spearman’s ranked test. For association between categorical variables, chi-square analyses were performed.

## Results

### A unique signature of dysregulated genes in peripheral blood mononuclear cells (PBMCs) of the tgDISC1 rat

**Fig. 2:**
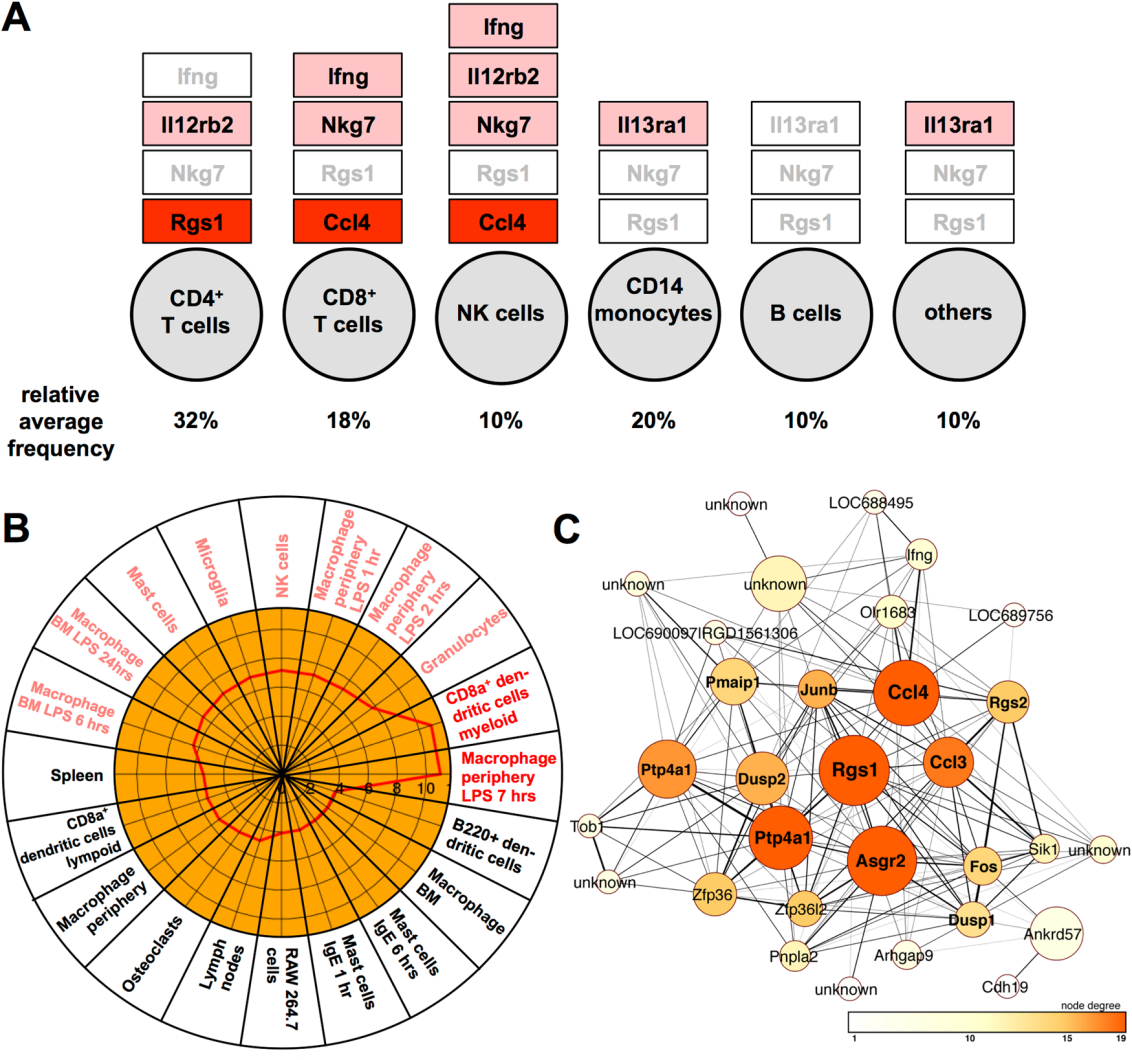
Assignment of top different transcripts of the tgDISC1 rat versus littermate control to peripheral blood mononuclear cell (PBMC) subtypes. **a** Top markers are mainly found in T cells and NK cells. Red: strong effects, light red: medium effects, white: weak effects. **b** A cell-type enrichment analysis (Cten; http://www.influenza-x.org/~jshoemaker/cten/)^63^ of the extended differential expression table (Supplementary Table 6) identified enrichment of differentially regulated transcripts in myeloid CD8^+^ dendritic cells, macrophages, granulocytes, NK cells, and microglia. See red line in inner circle for 1–10 fold enrichment. Red: strong association, light red: medium association, black: weak association. **c** Cytoscape illustration of the coexpression network with reduced expression levels in TG from the highly significantly deregulated “hotpink4” module after WGCNA. Nodes correspond to genes and edges connect co-expressed nodes with an adjacency value >0.2. Edge thickness corresponds to adjacency value. The node size is proportional to the stress centrality (importance) of the node, color represents the degree (number of edges) connectivity of the node from white (low) to dark orange (high)

In order to identify peripherally accessible, differentially expressed genes in our rat model for sporadic chronic mental illnesses, the tgDISC1 rat, we performed a microarray analysis of purified PBMCs from blood of tgDISC1, as well as wild-type littermate control rats. We identified various changes in gene expression, mostly downregulation, in PBMCs of the tgDISC1 rat (Table 1, see Supplementary Table 6 for an extended number). The top differentially expressed genes were regulator of G-protein signaling 1 (Rgs1) and chemokine (C–C motif) ligand 4 (Ccl4). Quantitative PCR of the target genes confirmed the expression changes in the transgenic rats (Supplementary Fig. S1). The top hits were reported to be expressed mainly in T cells and NK cells (Fig. 2a), and an analysis of the extended list of transcripts with at least a fold change of 1.2 as cutoff showed enrichment of genes expressed in activated macrophages, CD8^+^ myeloid dendritic cells, granulocytes, NK cells, and microglia (Fig. 2b).

**Table 1:** Top 20 genes differentially expressed in PBMCs of tgDISC1 rats compared with littermate controls

	Entry name	Gene symbol [rat]	Gene symbol [human]	Protein name	TG versus LM			qPCR performed	
					Change	FC	*P*	rat	human
1	RGS1_RAT	Rgs1	RGS1	Regulator of G-protein signaling 1	↓	2.03	0.0062	Y	Y
2	CCL4_RAT	Ccl4	CCL4	Chemokine (C–C motif) ligand 4	↓	1.67	0.0001	Y	Y
3	D4A8L8_RAT	Fpr2|Fpr2l	FPR2	Formyl peptide receptor 2 | Formyl peptide receptor 2-like	↓	1.65	0.0053	n.d.	Y
4	CO3_RAT	C3	C3	Complement component 3	↓	1.63	0.0037	Y	Y
5	Q9WVL9_RAT	Nkg7	NKG7	Natural killer cell group 7	↓	1.60	0.0004	Y	Y
6	F1LRH7_RAT	Il12rb2	IL12RB2	Interleukin 12 receptor, beta 2	↓	1.59	0.0095	Y	Y
7	ILEUA_RAT	Serpinb1a	SERPINB1	Serine proteinase inhibitor, clade B, member 1a	↓	1.52	0.0071	Y	Y
8	Q5MPU9_RAT	Ly49si3	–	Immunoreceptor Ly49si2	↓	1.51	0.0060	n.d.	n.d.
9	Q5M7T7_RAT	Pla2g7	PLA2G7	Phospholipase A2, group VII	↓	1.48	0.0056	n.d.	n.d.
10	H2A2A_RAT	LOC690131|Hist2h2aa3	HIST2H2AA3	Similar to H2A histone family, member O | Histone cluster 2, H2aa3	↓	1.47	0.0002	n.d.	n.d.
11	Q66HN6_RAT	Slc27a2	SLC27A2	Solute carrier family 27 (fatty acid transporter), member 2	↓	1.46	0.0089	Y	Y
12	Q561K3_RAT	Il13ra1	IL13RA1	Interleukin 13 receptor, alpha 1	↓	1.45	0.0074	Y	Y
13	CP4F3_RAT	Cyp4f18	CYP4F2	Cytochrome P450, family 4, subfamily f, polypeptide 18	↓	1.44	0.0038	n.d.	n.d.
14	RL10_RAT	Rpl10	RPL10	Ribosomal protein L10	↓	1.41	0.0059	n.d.	n.d.
15	D3ZPB4_RAT	Olr428	OR1L6	Olfactory receptor 428	↑	1.41	0.0092	n.d.	n.d.
16	IFNG_RAT	Ifng	IFNG	Interferon gamma	↓	1.38	0.0014	Y	Y
17	D1MF50_RAT	RGD 1561778	CD300C	Similar to dendritic cell-derived immunoglobulin-like receptor 1	↓	1.38	0.0055	n.d.	n.d.
18	F1LYV1_RAT	Scimp	SCIMP	SLP adaptor and CSK interacting membrane protein	↓	1.37	0.0018	n.d.	n.d.
19	TSN31_RAT	Tspan31	TSPAN31	Tetraspanin 31	↓	1.36	0.0041	n.d.	n.d.
20	D4AC93_RAT	Tmem223	TMEM223	Transmembrane protein 223	↓	1.35	0.0050	n.d.	n.d.

Arrow down, reduced expression in tgDISC1 rats, arrow up, increased expression in tgDISC1 rats. *FC* fold change, *qPCR* quantitative real-time PCR, *n.d.* not determined, *Y* qPCR performed (yes), *LM* non-transgenic littermate control, *TG* tgDISC1 rat

The gene ontology and ingenuity pathway analysis indicated enrichment of signaling pathways important for toll-like receptor signaling, T-cell activation and proliferation, communication between innate and adaptive immune response, and others (Table 2). In order to investigate which of our deregulated transcripts were part of coexpression networks and whether these networks were dysregulated in the tgDISC1 model compared with littermate controls, a weighted gene correlation network analysis WGCNA^37^ was performed (Supplementary Fig. S2). In this procedure, genes with similar expression patterns were classified into a total of 104 gene modules of which 10 were significantly altered in the tgDISC1 rat, with the module “hotpink4” displaying highest significance. Further analysis of the genes that are highly interconnected within that particular module by the Cytoscape NetworkAnalyzer indicated the microarray top hits Rgs1 and Ccl4 as functionally central hubs within that same module (Fig. 2c).

**Table 2:** Bioinformatic analysis of the microarray data (Supplementary Table 6) by Gene Ontology/Panther (top panel) or ingenuity pathway analysis (bottom panel)

Gene Ontology/Panther 13.1.	Raw	
*Name*	*P*	FDR
Positive regulation of toll-like receptor signaling pathway	1.14E-04	3.03E-02
Positive regulation of reactive oxygen species biosynthetic process	1.30E-04	3.23E-02
Cytokine production involved in immune response	2.08E-04	4.53E-02
Regulation of CD4-positive, alpha/beta T-cell activation	6.38E-05	2.00E-02
Positive regulation of cytokine production	1.16E-08	6.05E-05
Regulation of lymphocte proliferation	4.05E-05	1.47E-02
Positive regulation of ERK1 and ERK2 cascade	2.39E-04	4.80E-02

Ingenuity pathway analysis/top canonical pathways		
*Name*	*P*	Overlap
Communication between innate and adaptive immune cells	2.34E-05	4.3% 4/94
Role of hypercytokinemia in the pathogenesis of influenza	6.15E-05	7.0% 3/43
Altered T-cell and B-cell signaling in rheumatoid arthritis	5.52E-04	3.3% 3/90
Differential regulation of cytokine production in intestinal epithelial cells by IL17A and IL17F	7.65E-04	8.7% 2/23
Natural killer cell signaling	1.33E-03	2.5% 3/122

*FDR* false discovery rate

In order to analyze whether tgDISC1 rats exhibit an altered distribution of immune cell types, flow-cytometric phenotyping was performed, showing no significant differences between genotypes (Supplementary Fig. S3 for blood; S4 for splenocytes), although there was a strong trend toward increased T-cell number in tgDISC1 rats and significantly increased Cd3g expression, a pan T-cell marker, in PBMCs of the tgDISC1 rat (Supplementary Fig. S1I). Nevertheless, Cd3g level did not correlate with either Rgs1 or Ccl4 (Supplementary Fig. S1K) and an analysis of covariance confirmed that Cd3g is not a co-variate of Rgs1 [F(1,12) = 0.981, *p* = 0.342] or Ccl4 expression F(1,11) = 0.036, *p* = 0.853] in between-group analyses, which hints toward the fact that the reduction in transcript levels is due to a net reduced expression and not a reduction in the expressing cell-type number.

Taken together, these results indicate that the tgDISC1 rat displays a specific dysregulated network of immune-related genes.

### Genes differentially expressed in the tgDISC1 rat are similarly modulated in patients with schizophrenia

We next hypothesized that the PBMC gene expression changes in the tgDISC1 rat, as a model for subtly deranged DISC1-related signaling pathways, may reveal a similar signature of gene-expression changes in a subset of patients with schizophrenia where, potentially, signaling pathways of the extended DISC1 network are aberrant. We therefore first quantified the expression of the human orthologues corresponding to the top candidate genes deregulated in the tgDISC1 rat (listed in Table 1) by qPCR in a limited cohort of patients (group I^26,29^, for demographic information, see Supplementary Table 1) diagnosed with schizophrenia according to DSMIV/SCID and matched controls. In concurrence with this hypothesis, the top candidates RGS1, CCL4, and others were decreased and showed a similar pattern of regulation as observed in the tgDISC1 rat (Table 4; Supplementary Fig. S5A–F).

To corroborate the differential expression of these markers, we obtained samples from an independent and larger cohort of well-characterized schizophrenia cases and controls (group II, for demographic information, see Supplementary Table 1) that were tested blind with regard to diagnosis. Again, the top candidates RGS1, CCL4, and others were significantly decreased (Supplementary Figs. S5A–F, S6A–H). A summary of qPCR results of all targets tested in the tgDISC1 rat and the two independent human cohorts is provided in Table 3. We also tested whether RGS1, which is expressed by microglia, is differentially expressed in the brain samples of mental illness patients, but could not detect significant differences in patients and controls (Supplementary Table 5). CCL4 was not investigated, as it is not expressed in the brain.

**Table 3:** Overview table summarizing the different targets tested in rat and human cohorts

	Rat		Human qPCR	
Target	Microarray	qPCR	Group I	Group II
RGS1	↓ decrease	↓ decrease	↓ decrease	↓ decrease
CCL4	↓ decrease	↓ decrease	↓ decrease	↓ decrease
NKG7	↓ decrease	n.s.	n.s.	↓ decrease
C3	↓ decrease	↓ decrease	↓ decrease	n.s.
IL12RB2	↓ decrease	n.s.	n.s.	↓ decrease
DISC1	–	n.d.	n.s.	n.s.
IFNG	↓ decrease	↓ decrease	n.s.	n.s.
IL13RA1	↓ decrease	n.s.	n.s.	n.s.
SLC27A2	↓ decrease	n.s.	n.s.	n.s.
CCR5	↓ decrease	n.d.	n.s.	n.s.
SERPINB1	↓ decrease	n.d.	n.d.	n.s.
KMO	↓ decrease	n.d.	n.d.	↑ increase
FPR2	↓ decrease	n.d.	n.d.	↑ increase
JAK2	n.d.	n.d.	n.d.	n.s.
IL1B	–	n.d.	n.d.	n.s.
CD14	–	n.d.	n.d.	↑ increase
NKp46	–	n.d.	n.d.	↓ decrease
CD4	–	n.d.	n.d.	n.s.
CD8B	–	n.d.	n.d.	n.s.
CD3g	–	↑ increase	n.d.	n.d.
CD11b	–	n.s.	n.d.	n.d.

*n.s.* no significant difference, *n.d.* not determined

Arrow down marks a downregulation, arrow up an upregulation of the single targets in tgDISC1 rats or SCZ patients compared to the respective controls. Human samples are split for the two independent cohorts analyzed (group I and II)

Our findings regarding transcript levels were based on frozen human PBMCs that derive from a complex mixture of cell types. We therefore aimed to understand which cell type caused differential expression of the candidate marker genes in the absence of available flow-cytometry data for these same patients. We investigated the cell-type-specific expression of the marker genes by performing microfluidics-based single-cell RNA sequencing^34^ on ~3,000 human healthy donor-derived PBMCs. We also queried a published data set of ~8000 human PBMCs generated using a similar single-cell RNA-sequencing technique^42^ to validate the cell-type expression pattern in an independent dataset, as both derive from one donor. This combined approach confirmed the expression of the top genes in specific subpopulations of PBMCs (Supplementary Figs. S7, S8). We also used this data set to identify the most abundant cellular source of the marker genes. We found most abundant expression of the marker CCL4 in CD8^+^ T cells and natural killer (NK) cells and RGS1 with a predominance in CD4^+^ T cells even though present at low level in most cells (Fig. 2a; Supplementary Fig. S7A–D). This indicated that reduced expression of CCL4 could be due to its reduced expression by CD8^+^ T cells/NK cells, reduced abundance of CD8^+^ T cells/NK cell numbers in patients, or both. Similarly, RGS1 expression could correlate with cell abundance or expression in CD4^+^ T cells.

**Fig. 3:**
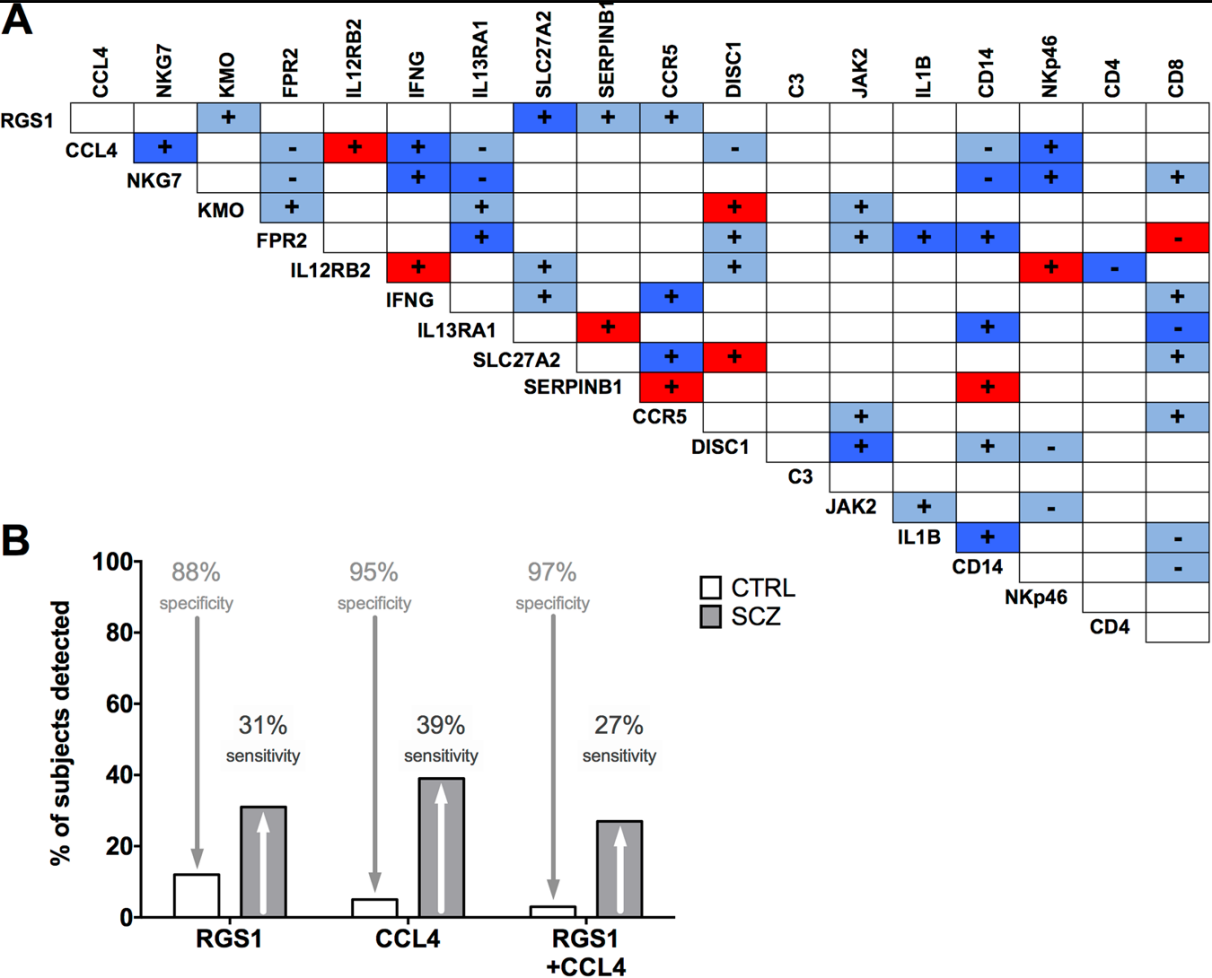
Non-correlating expression markers yield highest diagnostic specificity for schizophrenia patients. **a** The correlation matrix depicts present or absent co-regulated gene expression of the top hits in PBMCs derived from human schizophrenia patients and healthy controls. Individual expression levels were correlated using Spearman’s ranked test (group II). + stands for a positive, − for a negative correlation between target expression. Dark blue color indicates correlations that appear in both, SCZ and CTRL subjects; light blue color marks correlations seen only in CTRL subjects, i.e., that were lost in SCZ cases; correlations exclusively appearing in SCZ patients are depicted in red color, color-coding for physiological (light to dark blue) to more pathological (red) relations. All correlation coefficients, *P*-values and specific *n* can be found in Supplementary Fig. S9. **b** Specificity and sensitivity of potential biomarkers RGS1 and CCL4 in the detection of schizophrenia (SCZ) patients. Only cases that showed a target expression lower that 50% of the mean of control (CTRL) cases were counted as detected. By this analysis, information concerning sensitivity and specificity of the targets RGS1 and CCL4 could be gathered. By utilizing RGS1 levels alone, a subgroup of 31% of the SCZ cases could be detected, with a false positively detecting 12% of CTRL subjects. CCL4 analyzed in that manner identified a subgroup of 39% of SCZ patients with a specificity of 95% for detection of SCZ patients. A combination of both biomarkers led to a specificity of 97% and a sensitivity of 27%. CTRL control subjects, SCZ schizophrenia patients

To further address this issue, we performed qPCR for major markers of monocytes, T and NK cells (CD14, CD4, CD8, NKp46, respectively; Supplementary Fig. S5G–J), in which the top targets were most abundantly expressed in group II. Even though NK cell marker NKp46 was significantly reduced in SCZ patients of group II (Supplementary Fig. S5H), an analysis of covariance (ANCOVA) for the cell-type markers as co-variate was negative for RGS1 (CD4 F(1, 81) = 0.301, *p* = 0.585; CD8 F(1,82) = 0.146, *p* = 0.703; NKp46 F(1, 72) = 0.477, *p* = 0.492; CD14 F(1, 75) = 681.136, *p* = 0.583). For CCL4, solely NKp46 was found to be a co-variate (CD4 F(1, 79) = 0.025, *p* = 0.875; CD8 F(1, 80) = 1.486, *p* = 0.226; NKp46 F(1, 71) = 36.764, *p* < 0.001; CD14 F(1, 73) = 1.454, *p* = 0.232) and the expression of these two genes correlated (Fig. 3a), arguing that the expression change of CCL4 is also partially caused by a change in NKp46-positive cells that mainly comprises the NK cell population. T cells, however, are the most abundant cell type in PBMCs (about 50%), and NK cells comprise only around 10%^43^ (Fig. 2a), which makes the decrease of transcript levels likely due to their decreased expression in T cells. This result also leads to the conclusion that one single marker is not sufficient to serve as a biomarker but that expression changes of several genes, preferably from a single module in the identified dysregulated network (see Fig. 2c) should be taken into account (see also Discussion section).

We also analyzed expression of several other genes to further delineate the nature of the dysregulation of the network of immune-related genes in patients, potentially paralleling the one discovered in the tgDISC1 rat. In human PBMCs, DISC1 itself was overall expressed at low levels in blood cells with highest expression in CD14^+^ monocytes and memory T cells (Supplementary Fig. S7E, F). In our data, we found cell-type-specific expression patterns of the prion protein (PrP) comparable with that of DISC1 (Supplementary Fig. S7G–H), but much higher, as expected. This is a relevant control because DISC1 expression in the tgDISC1 rat is driven by the PrP promoter^16^. Furthermore, we also analyzed expression of interleukin (IL)-1β that has previously been reported as elevated in full blood samples of schizophrenia patients^44^, but IL-1β transcript levels in PBMCs measured by qPCR were unchanged (Supplementary Fig. S6I). Gene-expression profiles of the other targets tested in PBMCs subclasses can be found in Supplementary Fig. S8.

### A matrix of coregulated genes indicates that a specific, minimal combination of two markers defines a subset of ~25% of patients with schizophrenia with 97% specificity

The top markers identified in the tgDISC1 rat formed a highly expression-correlated network of genes in human PBMCs (Fig. 3a; Supplementary Fig. S9). Whereas many correlations in the regulation of expression levels were concordant in both, healthy controls and schizophrenia patients (dark blue in Fig. 3a), indicating a functionally maintained network of gene regulation, other correlation pairs of coexpression were disturbed, for example, when either coregulation existing in healthy controls was abolished in patients (light blue), or newly emerged in the disease condition (red). Thus, the markers identified in the tgDISC1 rat PBMCs are evidence of a profoundly disturbed gene-expression network also mirrored in PBMCs of patients with schizophrenia.

These results suggested that a particular combination of complementary markers characteristic for this dysregulated network could represent a signature for a biologically defined subset of schizophrenia. Therefore, measuring a selection of several markers from Table 1, Supplementary Table 6, or combinations thereof, would lead to a panel of markers similarly changed in the tgDISC1 rat and a schizophrenia subset, as evidence of aberrant signaling of a pathway with DISC1 protein involvement. Since investigating such a complex panel potentially poses problems for clinical practice, we set out to more specifically define a minimal marker combination discriminating a subset of patients with high specificity. We chose the two top markers RGS1 and CCL4 since they were both decreased in the tgDISC1 rat and the two human cohorts (Table 3), appear both as hub genes in the WGCNA analysis (Fig. 2c), and do not directly correlate in the correlation matrix of human PBMC gene expression (Fig. 3a), indicating that they could complement each other.

When an arbitrary threshold for the combined RGS1 and CCL4 expression level analysis was set to be below 50% of the mean level of RGS1 and CCL4 expressed in healthy controls, the combination of both genes resulted in a specificity of detection of 97% in identifying the PBMC samples of schizophrenia patients correctly (Fig. 3b). The sensitivity was about 27% meaning that the test is valid for a subset of clinically defined patients that comprises roughly a fourth of the clinically defined schizophrenia cases. RGS1 and CCL4 analyzed on their own also had a high specificity of detection alone but their combination may have diagnostic advantages (see the Discussion section).

### Validation of the RGS1/CCL4 marker combination by a correlation with clinical and cognitive endophenotypes

To further investigate the significance of the identified markers, PBMC gene-expression levels were analyzed with regard to clinical and cognitive variables obtained from the schizophrenia patients of group II. Gene-expression analysis revealed the positive marker combination (RGS1/CCL4) as defined in Fig. 3b in 13 patients (*n* = 13). Group comparisons between this marker-defined subset and the other, “marker-negative” SCZ patients (*n* = 37) showed no significant differences in their medical history, years of education, and cross-sectional chlorpromazine equivalents at the day of blood drawing (Table 4). The absence of a statistically significant correlation between cross-sectional chlorpromazine equivalents, i.e., the dosage of neuroleptic medication administered at the time of blood drawing, argues against an influence of medication on the levels of RGS1 and CCL4. Patients in the marker-negative group showed slightly more general symptoms (*p* = 0.035) as measured by the Positive and Negative Syndrome Scale (PANSS; Table 4). Comparison of means (Supplementary Table 2) revealed that the marker combination of RGS1 + CCL4 but not RGS1, CCL4, or DISC1 expression alone associated with better performance in the Digit Symbol Substitution Test (DSST, *p* = 0.027) that comprises testing for memory and attention. However, the lacking for multiple testing and the inequality of the sample sizes are potential caveats of this result. Further data regarding cognition and marker correlations can be found in Supplementary Table 3.

**Table 4:** Demographic and clinical characteristics of the marker-positive subgroup of cohort II

	No marker combination (*n* = 37)		Marker combination (*n* = 13)		F(df_1_, df_2_)	*P*
	M	(SD)	M	(SD)		
Age	32.24	(11.13)	40	(11.19)	4.66 (1, 48)	0.036^*^
Duration of illness	8.55	(9.04)	9.54	(8.33)	0.12 (1, 48)	0.732
Hospitalizations	4.03	(3.33)	3.15	(1.57)	1.54 (1, 43.31)	0.222
PANSS total	61.67	(17.50)	54.92	(12.44)	1.62 (1, 47)	0.209
PANSS positive	14.03	(5.59)	11.54	(3.57)	2.23 (1, 47)	0.142
PANSS negative	16.75	(5.12)	16.69	(6.58)	<0.01 (1, 47)	0.125
PANSS general	30.89	(9.31)	26.69	(4.11)	4.75 (1, 44.86)	0.035^*^

	No marker combination (*n* = 37)	Marker combination (*n* = 13)		
	Mdn	Mdn	U	*P*
CPZ	467.86	467.5	253.5	0.773
Age of onset	21	30	331	0.045^*^
Educational years	13	16	321.5	0.072

	No marker combination (*n* = 37)	Marker combination (*n* = 13)	χ^2^(df_1_)	*P*
Sex (male:female)	29:9	8:2	0.45 (1)	0.503
Clozapine users (yes:no)	5:32	8:5	3.74 (1)	0.053
Language (German:foreign)	30:7	13:0	2.86 (1)	0.091
Handedness (right:left:both)	33:4:0	11:1:1	2.96 (2)	0.227

*CPZ* chlorpromazine equivalents (cross-sectional at day of blood drawing), *PANSS* positive and negative syndrome scale, *M* mean, *SD* standard deviation, *Mdn* median, *df* degree of freedom, *F* F statistic, *U* Mann–Whitney-U, P *p*-value

Statistics represent the comparison of the patient groups with and without the marker combination

Pharmacological guidelines in Germany recommend usage of clozapine after unsuccessful therapeutic administration of at least two non-clozapine antipsychotics of different chemical classes^45^. Patients who receive clozapine are therefore likely non-responders to typical dopamine-2 receptor–antagonist pharmacotherapeutic treatment. In this study, patients with the positive marker combination showed a later age of onset of disease and higher proneness to receive clozapine treatment (Table 4; 8/13 marker-positive patients clozapine-treated vs. 5/37 marker-negative, non-clozapine-treated schizophrenia patients), albeit due to the small number of cases only at the level of a statistical trend at borderline significance (*p* = 0.053), suggesting that, potentially, the marker combination could relate to a biological subset less susceptible to typical neuroleptic pharmacotherapy (Table 4).

## Discussion

In this study, we identified a subset of schizophrenia patients (27%) characterized by a unique dysregulation of a network of immune-related genes, a delayed age of disease onset, as well as lighter positive, negative, and cognitive symptoms. The identification of this biologically definable schizophrenia subset was achieved by reverse translating (Fig. 1) PBMC transcripts identified as differentially expressed in tgDISC1 versus littermate control rats as an animal model for an aberrant signaling pathway with DISC1 protein involvement. This reverse-translational approach leads to a unique pairing of a patient subset, a biological diagnostic marker test, and a corresponding animal model that likely reflects aspects of an aberrant pathophysiology or brain signaling of that patient subset.

While we demonstrated that DISC1 overexpression and misassembly causes the specific dysregulation of immune-related gene networks in the tgDISC1 rat, among them RGS1 and CCL4 transcripts as top hits decreased in PBMCs, the argument that any marker-positive patient subset would display DISC1 protein pathology in their brains cannot be made so far. For that, we would need a collection of brain samples paired with PBMC samples, and no such collection is currently available as it is presently not feasible to visualize DISC1 protein pathology other than by biochemical fractionation of insoluble DISC1 protein from *post mortem* brain samples^9^.

Alternatively, it would be conceivable that in the RGS1/CCL4-positive schizophrenia patient subset, signaling pathways that involve the DISC1 protein are dysregulated in the brain without DISC1 protein misassembly but leading to similar changes in PBMC gene expression, either directly or indirectly through a neuro–immune interaction.

The combination of top markers RGS1/CCL4 was associated with deficits in cognitive functions in the DSST that have been repeatedly reported to be impaired in schizophrenia^46,47^. The subset detected by the marker combination also represents patients with a later onset and less reduced general PANSS score. At this point, we do not have an explanation why a dysfunctional RGS1/CCL4 signaling module, possibly linked to aberrant DISC1 signaling, led to the identification of patients with a less severe disease course. Nevertheless, we observed that the RGS1/CCL4 marker combination was overrepresented in clozapine users. This could be interesting for future, more rigorous investigations with much higher case numbers since a test for RGS1/CCL4 could eventually lead to the identification of patients non-responsive to typical dopamine 2-receptor antagonizing drugs, thus defining treatment resistance^48^. It is interesting to note that clozapine itself has immunomodulatory effects and that the role of these effects on the amelioration of schizophrenia symptoms has not been elucidated^49^. The absence of a positive correlation between the medication at the time of blood drawing and RGS1/CCL4 argues against an interpretation that the decreased levels of RGS1/CCL4 could be caused by psychotropic medication of the schizophrenia patients. Nevertheless, it cannot be excluded that among the vast number of different psychotropic medications some specific ones could modulate the expression of genes listed in Table 1 even though they were identified in unmedicated tgDISC1 rats.

We propose to apply testing for RGS1/CCL4 in many more cases of chronic mental illness, not limited to schizophrenia, but also to include recurrent affective disorders and neurological brain diseases to delineate its diagnostic accuracy. This is not only suggested by the pleiotropy of effects of DISC1 on clinical psychiatric phenotypes in particular^50^ but also by the pleiotropy of other genetic effects on diverse clinical diagnoses^4^. Furthermore, in order to provide diagnostic accuracy, inclusion of testing against immune-related brain diseases such as multiple sclerosis will be paramount. Various PBMC gene expression analyses on schizophrenia patients have been published^51–53^. Interestingly, a recent meta-analysis also identified significantly decreased CCL4 gene expression in schizophrenia patients, as well as other modulated genes from the same correlated network (see Fig. 2c), but not RGS1^53^. These gene expression (meta-) analyses were performed on groups of patients based on a clinical diagnosis which could dilute out any significant marker (combinations) in subsets. In the hypothesis-guided analysis presented here, however, a causal model, the tgDISC1 rat, was used to define aberrant PBMC signaling that was then rediscovered in a schizophrenia subset converging on the marker combination CCL4/RGS1.

It is remarkable that modest overexpression of DISC1 leads to such a profound dysregulation of immune functions (Table 2, Fig. 2). This opens the possibility that apart from the well-established functions of DISC1 in neurodevelopment and synaptic function^25^, DISC1 plays also a role in regulating the (neuro-) immune response. Whether in this function it may also contribute to mental illness by increasing susceptibility to pathogens or autoimmune responses remains to be investigated.

The top marker RGS1 is a regulator of G-protein signaling. It is a cytosolic protein located at the inner plasma membrane, transcribed from chromosome 1q31. RGS1 is the only reported gene significantly changed among the top markers that is also expressed in the brain, in microglia cells. On the genetic level, an RGS1 haplotype has been associated with depression and anxiety^54^. RGS4, another member of the RGS family of proteins but not with high homology in terms of sequence conservation, has been consistently associated to schizophrenia^55^. RGS1 single-nucleotide polymorphisms are also associated with multiple sclerosis^56,57^, but have not been detected in GWAS studies of schizophrenia^5^. It is interesting to note that a co-regulation database (CORD) analysis^58^ sees HLA genes DRA, DPA1, DRB1, and DQB1 as most highly co-regulated by RGS1. Thus, even though genetic evidence for involvement of RGS1 in “whole” schizophrenia comprising all clinical cases is sparse at this moment, a functional link to the major histocompatibility complex (MHC), the most consistently identified common variant in schizophrenia with the highest LOD score^5^, suggests that RGS1 itself could well play a so far underappreciated role in schizophrenia.

RGS1 has also been reported as a prognostic marker in melanoma or spondylarthritis^59,60^, indicating that there are roles for RGS1 in other disease conditions, even though in the schizophrenia subset its expression seems to be decreased rather than increased as in those other diseases. For mental illness diagnostics, it seems therefore justified to combine it with a complementary gene, to constitute a marker signature with high definition against those other disease conditions, as done here with CCL4.

One more reasoning argues in favor of a combination of genes as biomarkers, rather than one single marker alone. At this point, it is unclear whether in the schizophrenia patients investigated here, CCL4 expression is decreased in PBMCs or whether specific PBMC subpopulations accountable for its expression are decreased in number. CCL4 is mainly expressed in NK cells, CD8^+^ T cells, and CD14^+^ monocytes (Supplementary Fig. S6). NKp46, a NK cell marker, was decreased in cohort II (Supplementary Fig. S4J), and an analysis of covariance gave a significant influence of NKp46 level on CCL4 expression. One way to address the issue of cell number changes would be to apply flow-cytometry to patient blood. We did not have access to fresh blood samples and used frozen PBMCs that have been sampled over a period of time, but by a deconvolution approach, it has already been shown that the NK cell population is indeed reduced in schizophrenia, but not bipolar disorder patients^61^. Nevertheless, NK cells constitute only 10% of PBMCs, whereas, for example, CD8^+^ T cells and CD14^+^ monocytes constitute both around 30 and 20% of cells^43^, respectively, so the effect of NK cells on CCL4 expression will most likely be diluted out. Also, in the tgDISC1 rat, no change in NK cell levels, but nevertheless a significant decrease of CCL4 expression in PBMCs was detected. Thus, it seems of less importance whether one marker captures cell number effects, as long as the schizophrenia subset identified by that marker constitutes one clinically relevant entity, for example for predicting treatment resistance. Hence, it is an advantage to combine a minimum of two markers to rule out non-specific gene regulatory effects on marker expression.

As a soluble cytokine in plasma, CCL4 was previously found elevated in schizophrenia patients^62^, as were other proinflammatory cytokines, such as interleukin 1β^44^, which in this study was not changed when PBMC expression was investigated (Supplementary Fig. S5G). This is not in contradiction to our findings: transcript expression in cells and half-life time in plasma may follow a very different kinetics of degradation and, also, there may be subsets of patients with schizophrenia, such as the 27% identified here, that may actually divert from the mean.

In summary, this study has defined a subset of schizophrenia cases with a specific dysregulation of immune-related genes and particular clinical characteristics, inspired by a reverse-translational approach from an animal model. The main aim of this study was to show a novel and innovative way as well as a proof-of-concept to identify subsets of mental illness patients by reverse translation. Molecular details of DISC1 protein-related signaling pathways and immune-related genes will need further elaborations. While more studies are needed to characterize the RGS1/CCL4-defined schizophrenia subset and its clinical relevance, our here-proposed approach may prove valuable for changing the diagnostic culture in clinical psychiatry.

## Supplementary information

Supplementary Tables 1-6

Supplementary Table 6

Supplementary Figures S1-S9

## Data Availability

All data and materials presented here are available.
