# Supplementary material for: Dysregulation of a specific immune-related network of genes biologically defines a subset of schizophrenia
Source: Transl Psychiatry. 2019 May 31;9:156. doi: 10.1038/s41398-019-0486-6 (PMC6544656; doi:10.1038/s41398-019-0486-6)
Supplement: Supplementary file 1 — Supplementary Tables 1-6 [file 41398_2019_486_MOESM1_ESM.pdf]

Dysregulation of a specific immune-related network of genes biologically defines a subset of schizophrenia

Authors: Svenja V. Trossbach[1], Laura Hecher[1†], David Schafflick[2], René Deenen[3$], Ovidiu Popa[4], Tobias Lautwein[2,3], Sarah Tschirner[1], Karl Köhrer[3], Karin Fehsel[5], Irina Papazova[6], Berend Malchow[6], Alkomiet Hasan[6], Georg Winterer[5‡], Andrea Schmitt[6,7], Gerd Meyer zu Hörste[2], Peter Falkai[6], Carsten Korth[1*]

Contents: Supplementary Tables in order of appearance

Supplementary Tables

Supplementary Table 1: Demographic and clinical characteristics of schizophrenia patients and controls of cohort I and II.

Supplementary Table 2: Correlation analysis of top blood marker levels with neurocognitive variables.

Supplementary Table 3: Correlation analysis between further PBMC transcripts and cognitive domains or tests.

Supplementary Table 4: Primer information, cDNA dilution factor, PCR supplements and *n* depicted for the different human and rat target genes and cohorts.

Supplementary Table 5: Demographic and tissue information of SMRI Array Collection.

Supplementary Table 6: Extended microarray data tgDISC1 rat vs. controls as Excel-File (not included here, separate file)

Supplementary Tables in order of their appearance

Cohort I demographics

	CTRL			SCZ			
	N	**mean**	**SD**	***N***	**mean**	**SD**	***P***
age	50	34.18	11.74	16	34.31	8.45	0.691
PANSS Total				16	53.75	21.83	
PANSS General				16	26.75	11.89	
PANSS Positive				16	11.50	5.63	
PANSS Negative				16	15.50	6.15	
school years	49	11.82	1.39	16	11.43	1.41	0.336
professional school years	47	3.00	1.76	16	2.31	1.70	0.209

	**CTRL**	**SCZ**	$\chi^2(df_1)$	***P***
sex (male:female)	21:29	13:3	7.476 (1)	0.009*
education (1-4)	3:4:12:5:24	0:2:7:2:5	3.622 (4)	0.460
profession (1-5)	11:23:3:9:1	6:6:3:1:1	4.727 (4)	0.317

Cohort II demographics

	CTRL			SCZ				
	N	**M**	**(SD)**	***N***	**M**	**(SD)**	$F(df_1, df_2)$	***P***
age	45	30.24	9.34	50	34.26	11.554	6.106 (1, 93)	0.065
duration of illness				50	8.81	8.79		
age of onset				50	25.41	9.09		
CPZ				50	534.46	356.30		
hospitalizations				50	3.80	2.97		
PANSS Total				50	59.88	16.47		
PANSS General				50	29.78	8.42		
PANSS Positive				50	13.37	5.22		
PANSS Negative				50	16.73	5.47		
school years	45	12.46	1.10	50	11.48	2.22	15.167 (1, 93)	0.007*
years of training	45	16.87	2.70	50	14.07	4.11	2.801 (1, 93)	0.003*

	**CTRL**	**SCZ**	$\chi^2(df_1)$	***P***
sex (male:female)	29:16	39:11	2.139 (1)	0.175
handedness (left:right:both)	5:39:1	5:44:1	0.038 (2)	0.981
language (german:foreign)	38:7	43:7	0.046 (1)	0.831
Clozapine users (yes:no)		40:10		

Supplementary Table 1: Demographic and clinical characteristics of the SCZ patients and controls of cohort I (upper two panels) and II (lower two panels). Statistics represent the comparison of SCZ patient groups with controls. Abbreviations: PANSS, Positive and Negative Syndrome Scale; CPZ, chlorpromazine equivalents (cross-sectional at day of blood drawing); education: 1 = no school degree, 2 = lowest school level “Hauptschule”, 3 = middle school level “Realschule”, 4 = highest school degree “Abitur”; profession: 1 = no, 2 = training, 3 = lower university degree “Fachhochschule”, 4 = university degree, 5 = PhD; M, mean; SD, standard deviation; Mdn, mean; df, degree of freedom; F, F statistic; χ^2, Chi Square; P, p-value.

		RGS1	CCL4	RGS1+CCL4		DISC1
	r	0.165	-0.149	0.085	***τ***	0.023
**VLMT: sum 1-5 (score)**	***p***	0.241	0.308	0.560	***p***	0.816
	N	52	49	49	***N***	49
	r	0.128	-0.149	-0.038	***τ***	0.04
**VLMT: trial 5 minus trial 7**	***p***	0.368	0.308	0.795	***p***	0.701
	N	52	49	49	***N***	49
	r	0.130	-0.044	0.139	***τ***	0.028
**TMT: A (in s)**	***p***	0.360	0.763	0.341	***p***	0.782
	N	52	49	49	***N***	49
	r	0.179	-0.190	0.003	***τ***	0.063
**TMT: B (in s)**	***p***	0.209	0.196	0.981	***p***	0.528
	N	51	48	48	***N***	48
	r	0.259	0.079	**0.313**	***τ***	0.076
**DSST**	***p***	0.062	0.584	**0.027***	***p***	0.441
	N	53	50	50	***N***	50
	r	0.199	-0.141	0.069	***τ***	0.004
**Memory domain**	***P***	0.162	0.339	0.640	***p***	0.965
	N	51	48	48	***N***	48
	r	0.230	-0.040	0.201	***τ***	0.028
**Attention domain**	***p***	0.101	0.784	0.165	***p***	0.776
	N	52	49	49	***N***	49
	r	0.216	-0.058	0.187	***τ***	-0.02
**Global cognitive index**	***p***	0.131	0.700	0.208	***p***	0,84
	N	50	47	47	***N***	47
	τ	-0.007	0.031	-0.004	***τ***	-0.111
**DST: forward (score)**	***p***	0.942	0.771	0.972	***p***	0.297
	N	52	49	49	***N***	49
	τ	0.195	-0.070	0.063	***τ***	-0.103
**DST: backwards (score)**	***p***	0.059	0.509	0.556	***p***	0.338
	N	52	49	49	***N***	49

Supplementary Table 2: Correlation analysis of top blood marker levels with neurocognitive variables in cohort II. Abbreviations: VLMT: Verbal Learning and Memory Test; DST: Digit-Span-Test; DSST: Digit Symbol Substitution Test; TMT: Trial Making Test; *r:* Pearson's correlation coefficient*; τ:* Kendall-Tau's correlation coefficient*, p*: p-value * $p < .05$

		NKG7	IL13RA1	IFNG	SLC27A2	IL12RB2	FPR2	KMO	SERPINB1	CCR5	C3	JAK2
	r	0.047	-0.041	-0.120	0.002	0.009	-0.093	-0.103	-0.161	-0.084	0.178	0.069
**VLMT: sum 1-5 (score)**	***p***	0.744	0.776	0.397	0.991	0.953	0.531	0.480	0.281	0.576	0.254	0.649
	N	50	50	52	52	49	48	49	47	47	43	46
	r	0.090	-0.065	-0.097	0.001	-0.160	0.284	0.141	-0.043	0.107	0.252	0.098
**VLMT: trial 5 minus trial 7**	***p***	0.534	0.654	0.494	0.996	0.272	0.050	0.334	0.776	0.475	0.103	0.518
	N	50	50	52	52	49	48	49	47	47	43	46
	r	0.071	0.022	0.134	0.028	0.275	-0.092	-0.021	-0.123	0.273	0.070	0.151
**TMT: A (in s)**	***p***	0.625	0.879	0.344	0.846	0.056	0.530	0.889	0.411	0.063	0.653	0.318
	N	50	50	52	52	49	49	49	47	47	44	46
	r	0.005	0.083	-,279*	-0.113	0.066	-0.132	0.045	-0.114	0.112	-0.126	0.212
**TMT: B (in s)**	***p***	0.975	0.569	**0.047***	0.429	0.658	0.372	0.760	0.451	0.458	0.420	0.163
	N	49	49	51	51	48	48	48	46	46	43	45
	r	0.214	-0.025	0.129	0.109	,392**	-0.125	-0.153	0.102	0.197	0.206	0.199
**DSST**	***p***	0.131	0.859	0.358	0.437	0.005	0.391	0.290	0.492	0.180	0.180	0.179
	N	51	51	53	53	50	49	50	48	48	44	47
	r	0.094	-0.021	-0.146	-0.080	-0.075	0.076	0.067	-0.151	0.050	0.227	0.021
**Memory domain**	***p***	0.520	0.888	0.307	0.575	0.611	0.610	0.653	0.316	0.741	0.147	0.890
	N	49	49	51	51	48	47	48	46	46	42	45
	r	0.136	0.026	-0.001	0.008	,309*	-0.133	-0.076	-0.037	0.244	0.080	0.238
**Attention domain**	***p***	0.347	0.858	0.992	0.956	0.031	0.363	0.606	0.805	0.098	0.605	0.111
	N	50	50	52	52	49	49	49	47	47	44	46
	r	0.134	0.031	-0.119	-0.077	0.167	-0.017	-0.058	-0.041	0.102	0.174	0.159
**Global cognitive index**	***p***	0.363	0.836	0.410	0.594	0.263	0.912	0.700	0.790	0.504	0.271	0.302
	N	48	48	50	50	47	47	47	45	45	42	44
	τ	-0.058	0.021	0.066	-0.114	0.079	0.022	0.023	-0.100	0.004	-0.021	0.016
**DST: forward (score)**	***p***	0.578	0.837	0.519	0.266	0.453	0.835	0.825	0.353	0.970	0.856	0.884
	N	50	50	52	52	49	48	49	47	47	43	46
	τ	0.096	0.030	0.070	-0.005	0.031	-0.046	-0.042	-0.119	0.106	0.192	0.103
**DST: backwards (score)**	***p***	0.362	0.776	0.499	0.961	0.769	0.672	0.690	0.274	0.327	0.092	0.352
	N	50	50	52	52	49	48	49	47	47	43	46

Supplementary Table 3: **Correlation analysis between further PBMC transcripts and cognitive domains or tests.** Abbreviations: VLMT: Verbal Learning and Memory Test; DST: Digit-Span-Test; DSST: Digit Symbol Substitution Test; TMT: Trial Making Test; *r:* Pearson's correlation coefficient*; τ:* Kendall-Tau's correlation coefficient, *p*: p-value $^{*}p < .05$

target (rat)	primer forward 5'-3'	primer reverse 5'-3'	primer conc F/R	cDNA dilution	add-ons	LM [n]	TG [n]
Actin	GAGAGGGAAATCGTGCGTG	CATGGATGCCACAGGATTCC	300/300 nM	dependent on target	no		
C3	GAGAGCTGGTTGTGGACCAT	CAGTCGCAGGTCAATGAAGA	50/300 nM	1:25	5% Factor Q	7	5
Ccl4	CTCTCTCCTCCTGCTTGTGG	CACAGATTTGCCTGCCTTTT	900/50 nM	1:25	5% Factor Q	8	6
Cd11b	TATTCGGCTCCAACCTGCTG	CGATCGTGTTGATGCTACCG	300/300 nM	1:10	no	8	7
Cd3g	CTGGACAGGATGGAGTTCGC	TTGGAGACGGCTGTACTGTTC	300/300 nM	1:10	no	8	7
Ifng	GCCCTCTCTGGCTGTTACTG	CTGATGGCCTGGTTGTCTTT	50/300 nM	1:25	5% Factor Q	7	3
Il12rb2	AGCCTCTTAACAGCACATCCT	TGAAATTCATATTCTGTGAATGGTCT	300/300 nM	1:25	no	8	5
Il13ra1	GAAACATGGAGGGTGCAAGT	CACTGCGACAAAGACTGGAA	300/300 nM	1:25	5% Factor Q	7	5
Il1b	CGTGGGATGATGACGACCTG	GCCACAGGGATTTTGTCGTT	300/300 nM	1:10	no	8	7
Nkg7	GAGCCCTACCGGTCCCTG	GCAGACCTGTGTCACGTGG	900/50 nM	1:10	no	8	7
Rgs1	CCTTGCCAACCAGATGGGTC	CAGCATCTGAGTGCACAAATGC	50/300 nM	1:10	no	8	7
Slc27a2	GCAGGAAATACAACGCCACT	TCTTCCAACAGCTCCGATTT	50/300 nM	1:25	5% Factor Q	8	5

						group 2 | LMU		group 1 | HHU	
target (human)	primer forward 5'-3'	primer reverse 5'-3'	primer conc F/R	cDNA dilution	add-ons	CTRL [n]	SCZ [n]	CTRL [n]	SCZ [n]
ARF1	GACCACGATCCTCTACAAGC	TCCCACACAGTGAAGCTGATG	300/300 nM	dependent on target	no				
ACTINB1	AGATCAAGATCATTGCTCCTCC	CTTGTTTTCTGCGCAAGTTAGG	300/300 nM	dependent on target	no				
C3	GCTCCAGACACAGATGACCTG	GCGTAGACCTTGACTGCTCCAG	50/300 nM	1:10	no	- - -	- - -	44	15
C3	CCCTCACGGCCTTTGTTCTC	GCCAGAGCATAGCCAGCAATG	300/300 nM	1:10	no	38	45	- - -	- - -
CCL4	CTGAGTTCTGCAGCCTCACC	CTGGGATCAGCACAGACTTGC	300/300 nM	1:10	no	41	51	49	15
CCR5	GAGACATCCGTTCCCCTACAAG	GTGAGTAGAGCGGAGGCAGG	300/300 nM	1:10	no	38	48	30	12
CD14	GGAAGACTTATCGACCATGGAGC	CGCAGCGGAAATCTTCATCG	300/300 nM	1:10	no	36	47	- - -	- - -
CD4	ATGAACCGGGGAGTCCCTTT	CAGCTTGGATGGACCTTTAGTT	300/300 nM	1:25	no	39	50	- - -	- - -
CD8B	CGCAGCTGACAGTTCTCCAT	GTCACTGCTCGGTGCCTG	300/300 nM	1:50	no	39	50		
DISC1	ACACCCCACTGAGAATGGAG	GTTGCTGCTCTTGCTCCTCT	300/300 nM	1:10	no	44	49	50	16
FPR2	GTGTCCTATGAGTCTGCTGG	CCATGGCCATGGAGACAATG	300/300 nM	1:10	no	45	50	- - -	- - -
IFNG	GGCTGTAGATTCTCGAGTGCGG	CGCTACATCTGAATGACCTGC	300/300 nM	1:50	no	43	54	50	14
IL12RB2	GACTGTGGCCTGCACCTG	GACAGCAGTAACCTTGGCTGTG	300/300 nM	1:10	no	42	51	47	16
IL13RA1	CCACCCGAGGGAGCCAGCTC	CTTCTGGGGGTGAGATGC	50/50 nM	1:10	no	44	52	50	16
IL1B	CAGAAGTACCTGAGCTCGCC	AGATTCGTAGCTGGATGCCG	300/300 nM	1:10	no	34	43	- - -	- - -
JAK2	GAATGTCTTGGGATGGCAGTG	CAGTGGCTTTGCATTGGCTG	300/300 nM	1:10	no	38	47	- - -	- - -

KMO	GAATGCGGGCTTTGAAGACTG	CGCGTGATCATCTGGGATTC	300/300 nM	1:10	no	44	50	- - -	- - -
NKG7	CCCGCTTGTCTCAACCACC	CACAGTGAGCACCCAGGC	300/300 nM	1:10	no	40	52	45	16
NKp46	GAAGCCTTTTTGCCGTGGAC	CAGGATGAACCGAGAGGGTG	300/300 nM	1:10	no	34	45	- - -	- - -
RGS1	GAGTTCTGGCTGGCTTGTGAAG	GGCTGTAGATTCTCGAGTGCGG	300/300 nM	1:10	no	43	54	49	16
SERPINB1	GACCAGAGGTAACACGGCAG	CACTGGCCAGGTCAGCAC	300/300 nM	1:10	no	39	48	- - -	- - -
SLC27A2	CCACGACAGAGTTGGAGATAC	GGCCTTGCATAACTAGGTAGG	300/300 nM	1:25	no	43	54	48	16

Supplementary Table 4: Primer information, cDNA dilution factor, PCR supplements and *n* depicted for the different human and rat target genes and cohorts. Abbreviations: LM, non-transgenic littermate control; TG, tgDISC1 rat; CTRL, control subjects; SCZ, schizophrenic subjects.

	total	expression	no measureable expression	no expression
	n	*n*	*n*	% of total
**CTRL**	7	7	0	0
**BD**	7	4	3	43
**SCZ**	8	7	1	13
P (χ2)	0.101			

	**N | control**			**B | Bipolar**			**S | Schizophrenia**		
	n	mean	SD	*n*	mean	SD	*n*	mean	SD
age	7	45.43	5.91	7	47.43	13.64	8	36.75	10.44
PMI	7	31.71	16.17	7	38.71	16.39	8	29.25	9.10
pH	7	6.60	0.23	7	6.34	0.43	8	6.51	0.17
Duration of illness				7	15.29	6.55	8	16.25	11.51
Age of onset				7	32.14	12.02	8	20.50	5.71
AP dose				7	2571	3552	8	91887	89126
RQN	7	8.36	0.52	7	8.44	0.29	8	8.36	0.49

	**N | control**	**B | Bipolar**	**S | Schizophrenia**
sex (male:female)	4:3	2:5	5:3
Drug Lifetime (1-6)	7:0:0:0:0:0	4:0:0:2:0:1	3:0:1:1:0:2
Alcohol Lifetime (1-6)	5:1:0:1:0:0	2:3:0:1:0:1	4:2:1:0:0:1

Supplementary Table 5: RGS1 expression in the brains of patients and controls of the SMRI Array Collection. RGS1 is expressed by microglia in the brain. Upper table shows number of patients being RGS1 expressors or not. No significant difference between groups could be detected by Chi Square analysis. Lower panels depict demographics and tissue information for the patient samples used. Abbreviations: PMI, post mortem interval; AP dose, lifetime antipsychotic treatment dose, RQN, RNA quality score (1-10, 10 best); drug and alcohol lifetime: 1 = none, 2 = social, 3 = moderate past, 4 = moderate present; 5 = heavy past, 6 = heavy present, χ^2, Chi Square.
