# Supplementary material for: Dysregulation of a specific immune-related network of genes biologically defines a subset of schizophrenia
Source: Transl Psychiatry. 2019 May 31;9:156. doi: 10.1038/s41398-019-0486-6 (PMC6544656; doi:10.1038/s41398-019-0486-6)
Supplement: Supplementary file 3 — Supplementary Figures S1-S9 [file 41398_2019_486_MOESM3_ESM.pdf]

Dysregulation of a specific immune-related network of genes biologically defines a subset of schizophrenia

Authors: Svenja V. Trossbach[1], Laura Hecher[1†], David Schafflick[2], René Deenen[3$], Ovidiu Popa[4], Tobias Lautwein[2,3], Sarah Tschirner[1], Karl Köhrer[3], Karin Fehsel[5], Irina Papazova[6], Berend Malchow[6], Alkomiet Hasan[6], Georg Winterer[5‡], Andrea Schmitt[6,7], Gerd Meyer zu Hörste[2], Peter Falkai[6], Carsten Korth[1*]

Contents: Supplementary Figures in order of appearance

Supplementary Figure S1: Validation of microarray hits deregulated in PBMCs of tgDISC1 rats compared to control rats by quantitative PCR.

Supplementary Figure S2: WGCNA analysis

Supplementary Figure S3: Flow cytometric phenotyping of tgDISC1 and littermate control blood.

Supplementary Figure S4: Flow cytometric phenotyping of tgDISC1 and littermate control splenocytes.

Supplementary Figure S5: Validation of microarray hits deregulated in PBMCs of tgDISC1 rats by qPCR in human PBMCs derived from two independent cohorts of patients diagnosed with schizophrenia and control subjects.

Supplementary Figure S6: Validation of further microarray hits deregulated in PBMCs of tgDISC1 rats by qPCR in human PBMCs derived from two independent cohorts of patients diagnosed with schizophrenia and control subjects.

Supplementary Figure S7: Expression profile of targets in human PBMC subclasses.

Supplementary Figure S8: Further expression profile data of targets in human PBMC subpopulations.

Supplementary Figure S9: Detailed correlation parameters between top hits expression levels measured in human subject samples of group II.

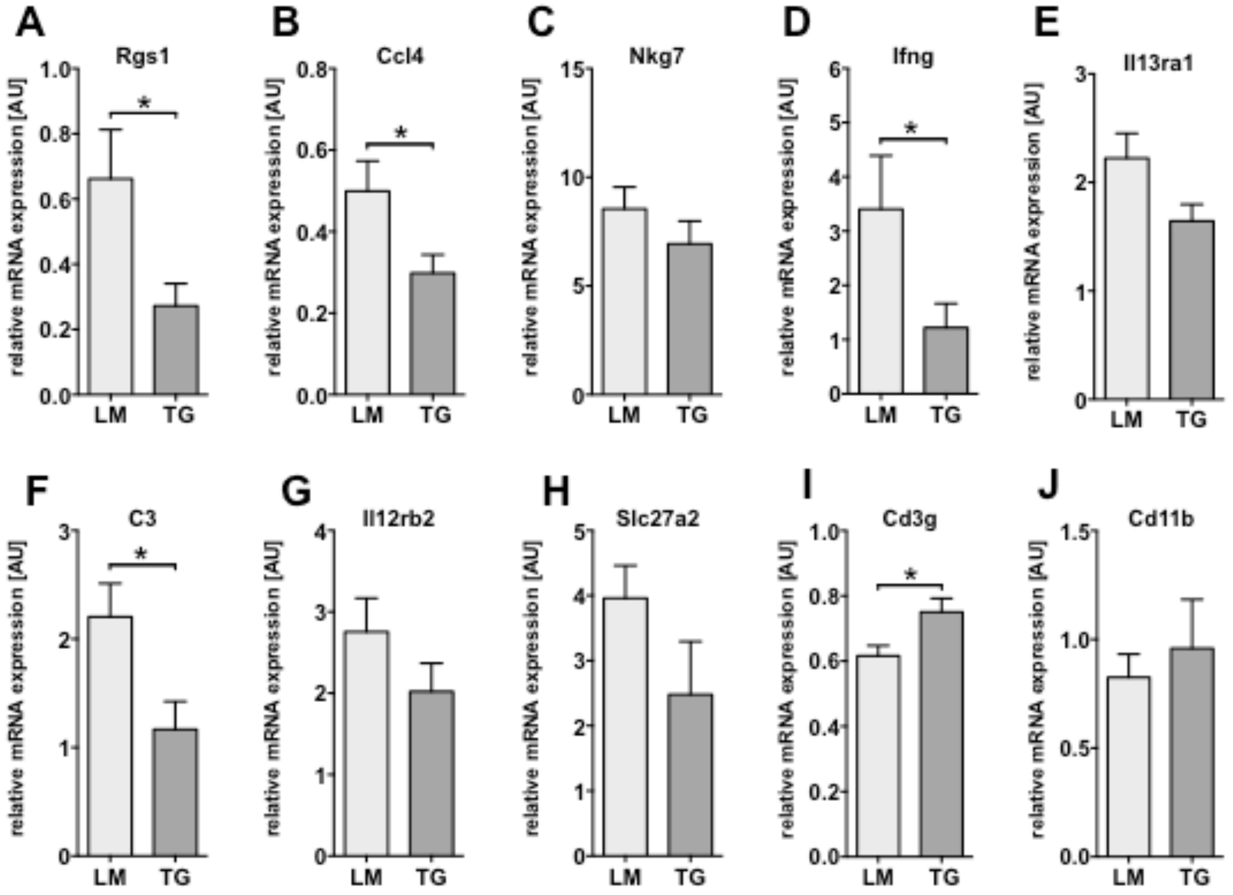

K

Cd3g			Rgs1	Ccl4	Nkg7	Ifng	Il 13ra1	C3	Il 12rb2	Slc27a2
Cd3g	TG	R	0.126	0.036	-0.286	0.5	-0.7	-0.8	-0.7	-0.6
		P	0.788	0.939	0.535	0.667	0.188	0.104	0.188	0.285
		N	7	7	7	3	5	5	5	5
	LM	R	0,19	-0,643	-0,381	-0,071	0,071	-0,107	0,167	0.0
		P	0.651	0.086	0.352	0.879	0.879	0.819	0.693	1.000
		N	8	8	8	7	7	7	8	8

Supplementary Figure S1: Validation of microarray hits deregulated in PBMCs of tgDISC1 rats (grey bars) compared to control rats (light bars) by quantitative Real-Time PCR. (**A**) Expression of Rgs1 was reduced in tgDISC1 rats. T-test $*P = 0.043$. (**B**) Relative mRNA levels of Ccl4 were reduced in tgDISC1 rats. T-test $*P = 0.043$. (**C**) No expression change of Nkg7 was detectable in tgDISC1 rats. T-test $P = 0.290$. (**D**) Reduced

levels of Ifng mRNA were found in tgDISC1 rats. Mann-Whitney U-test *P = 0.053. (**E**) A trend towards reduced expression of Il13ra1 was seen in tgDISC1 rats. T-test P = 0.085. (**F**) C3 mRNA levels were reduced in tgDISC1 rats. T-test *P = 0.035. (**G**) No change expression could be seen for Il12rb2 in tgDISC1 rats. T-test P = 0.244. (**H**) Slc27a2 was not differentially expressed PBMCs of tgDISC1 rats. T-test P = 0.129. (**I**) An increase in Cd3g mRNA hinting towards increased T-cell number could be detected in tgDISC1 rats. T-test *P = 0.022 (**J**) No change in Cd11b mRNA, a marker for monocytes, was detected in tgDISC1 rats compared to littermate controls. T-test P = 0.585. (**K**) As tgDISC1 rats showed increased expression of Cd3g, a pan T cell marker, the top hits were correlated against Cd3g data. No correlation of Rgs1 or Ccl4 levels with Cd3g could be found. Depicted are Spearman correlations with correlation coefficient R, number of animals N and P-value. Abbreviations: LM, non-transgenic littermate controls; TG, tgDISC1 rats; AU, arbitrary units. All means ± s.e.m.

A

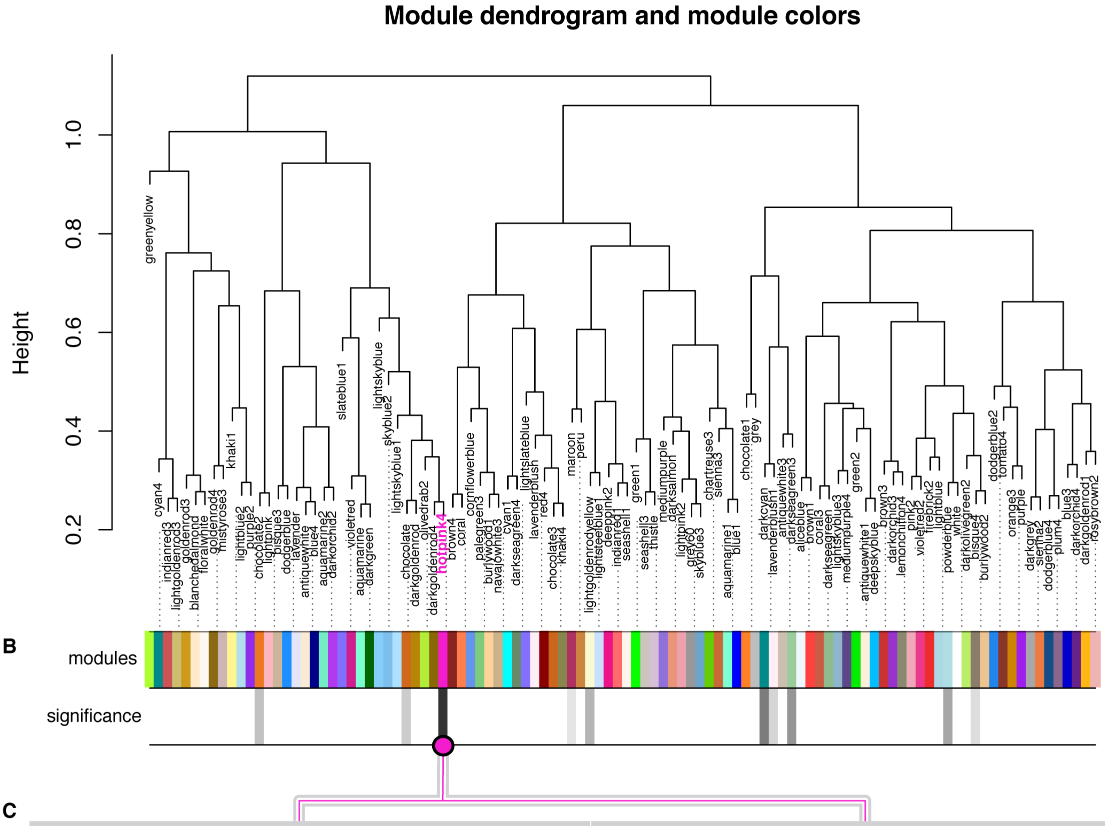

C

Coexpressed genes with lower expression level in the tgDISC1 samples

Transcripts Cluster Id	Gene symbol [rat]	Module Membership correlation	Module Membership p-value	Gene Trait correlation	Gene Trait p-value	Intramodule connectivity	average correlation (Pearsons)
17859412	**Ptp4a1**	-0.92	2.2E-05	-0.81	1.3E-03	0.88	0.29
17659402	**Ccl3**	-0.94	5.4E-06	-0.65	2.3E-02	0.93	0.28
17647707	**Asgr2**	-0.92	1.8E-05	-0.74	5.7E-03	0.92	0.27
17684919	**Rgs1**	-0.91	3.5E-05	-0.69	1.3E-02	0.91	0.26
17649604	**Ccl4**	-0.87	2.4E-04	-0.85	5.2E-04	0.70	0.26
17728638	**Junb**	-0.92	2.5E-05	-0.63	2.9E-02	0.88	0.25
17817508	**Fos**	-0.94	4.7E-06	-0.65	2.2E-02	1.00	0.24
17684906	**Rgs2**	-0.91	3.6E-05	-0.65	2.3E-02	0.85	0.24
17819882	**Zfp36l2**	-0.90	6.3E-05	-0.61	3.4E-02	0.86	0.24
17863629	**Ptp4a1**	-0.88	1.9E-04	-0.85	5.2E-04	0.71	0.24

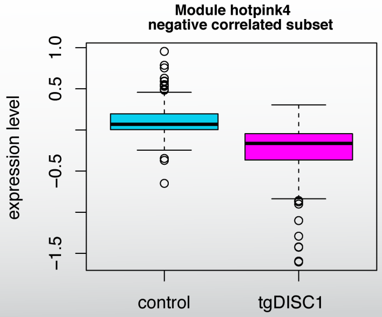

Coexpressed genes with elevated expression level in the tgDISC1 samples

Transcripts Cluster Id	Gene symbol [rat]	Module Membership correlation	Module Membership p-value	Gene Trait correlation	Gene Trait p-value	Intramodule connectivity	average correlation (Pearsons)
17619842	**unknown**	0.90	7.8E-05	0.73	6.9E-03	0.74	0.16
17629135	**Zik1**	0.89	1.3E-04	0.74	5.6E-03	0.72	0.16
17733013	**Kiaa0895l**	0.90	5.8E-05	0.71	9.9E-03	0.78	0.15
17837495	**Klhl38**	0.86	3.4E-04	0.76	3.8E-03	0.64	0.15
17691263	**unknown**	0.87	2.0E-04	0.67	1.6E-02	0.75	0.14
17749443	**Anxa9**	0.77	3.3E-03	0.85	4.2E-04	0.46	0.13
17690129	**RGD1562755**	0.88	1.4E-04	0.64	2.6E-02	0.70	0.13
17816034	**Mia2**	0.81	1.3E-03	0.72	7.7E-03	0.57	0.13
17636095	**LOC499240**	0.79	2.4E-03	0.76	4.5E-03	0.53	0.13
17766456	**Mir103-2**	0.82	1.1E-03	0.74	5.7E-03	0.58	0.13

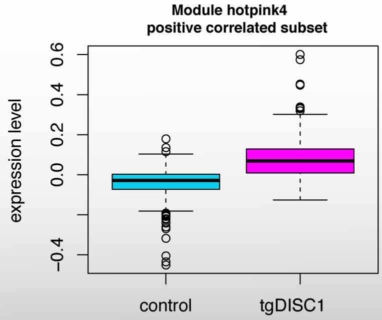

Supplementary Figure S2: Co-expression analysis using WGCNA approach. (**A**) Dendrogram of 104 modules calculated using the dissimilarity of the module eigengenes. (**B**) A color code is assigned for each module (color bar modules) and each module has an indication if it is significant regarding clinical traits (color bar significance). Non-significant

modules are white, modules with p-value <0.05 are colored in grey shades depending on their significance from light grey to black which is the most significant module (hotpink4). (**C**) Top 10 of most interconnected genes within the hotpink4 module divided into reduced expression level of TG compared to LM (left side) and elevated expression levels of TG compared to LM (right side). The differences in expression levels between TG and LM are shown in the box plot at the bottom of each subset. Abbreviations: LM, non-transgenic littermate controls; TG, tgDISC1 rats.

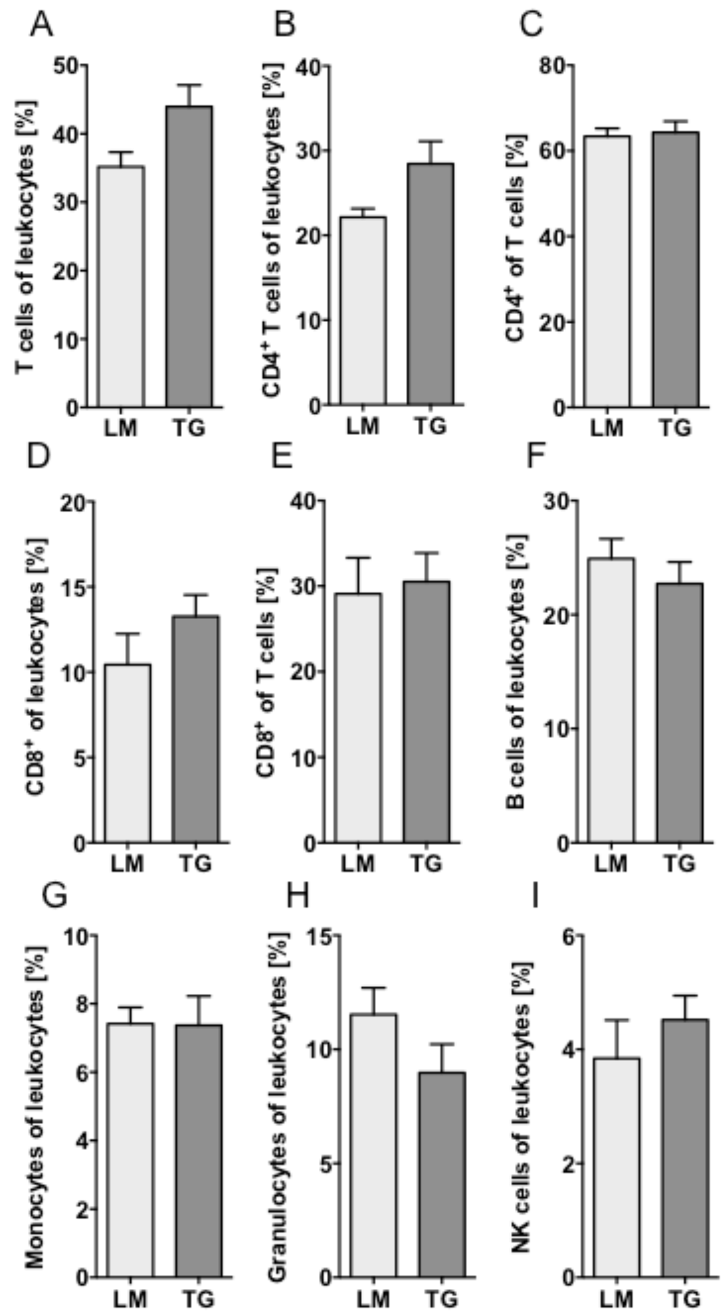

Supplementary Figure S3: Flow cytometric phenotyping of tgDISC1 and littermate control blood. TgDISC1 rats and littermate controls were immunologically phenotyped by flow cytometric analysis of common leukocyte lineages in the blood. (**A**) TgDISC1 rats showed a strong trend towards increased numbers of T cells of leukocytes. T-test $P = 0.052$. (**B**) The $CD4^+$ T cell population of leukocytes was slightly increased in tgDISC1 rats. T-test $P = 0.076$. (**C**) The $CD4^+$ T cell population of T cells was comparable between tgDISC1 rats

and controls. T-test *P* = 0.784. (**D**) The number of $CD8^+$ cells of leukocytes was unchanged. T-test *P* = 0.232. (**E**) There was no difference in percentage of $CD8^+$ T cells of leukocytes. T-test *P* = 0.797. (**F**) B cells were comparable between tgDISC1 rats and controls. T-test *P* = 0.416. (**G**) There was no difference in monocyte populations. T-test *P* = 0.963. (**H**) Numbers of granulocytes were unchanged between animal groups. T-test *P* = 0.170. (**I**) TgDISC1 rats had comparable NK cell population compared to controls. T-test *P* = 0.415. Abbreviations: LM, non-transgenic littermate controls; TG, tgDISC1 rats. All means ± s.e.m.

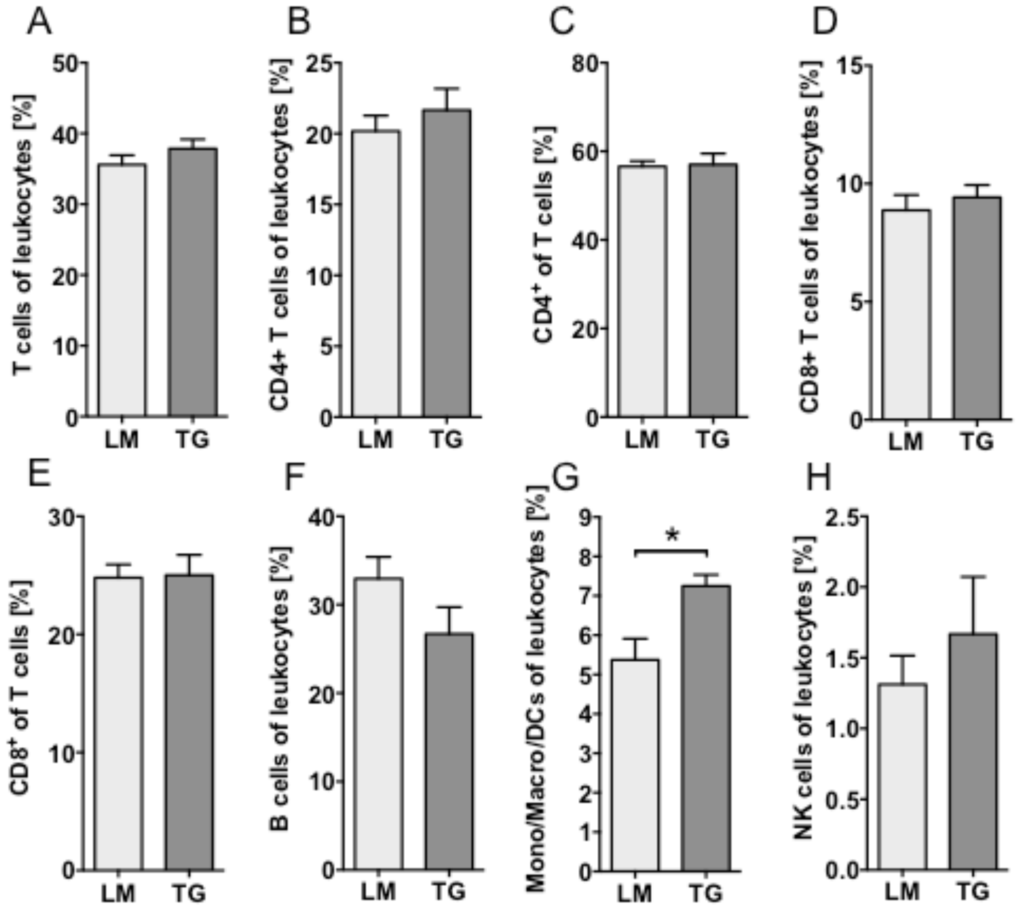

Supplementary Figure S4: Flow cytometric phenotyping of tgDISC1 and littermate control splenocytes. (**A**) Numbers of T cells of leukocytes are comparable between tgDISC1 and littermate control rats. T-test $P = 0.259$. (**B**) TgDISC1 rats had comparable $CD4^+$ T cell populations of leukocytes. T-test $P = 0.450$. (**C**) Also the number of $CD4^+$ T cells of leukocytes was unchanged. T-test $P = 0.879$. (**D**) There was no difference in percentage of $CD8^+$ T cells of leukocytes. T-test $P = 0.523$. (**E**) The number of $CD8^+$ cells of T cells was unchanged. T-test $P = 0.917$. (**F**) There was no difference in B cell populations. T-test $P = 0.150$. (**G**) TgDISC1 rats show an increase in monocyte/macrophage/dendritic cell populations. T-test $*P = 0.021$. (**H**) Numbers of NK cell populations were unchanged between animal groups. T-test $P = 0.461$. All means ± s.e.m.

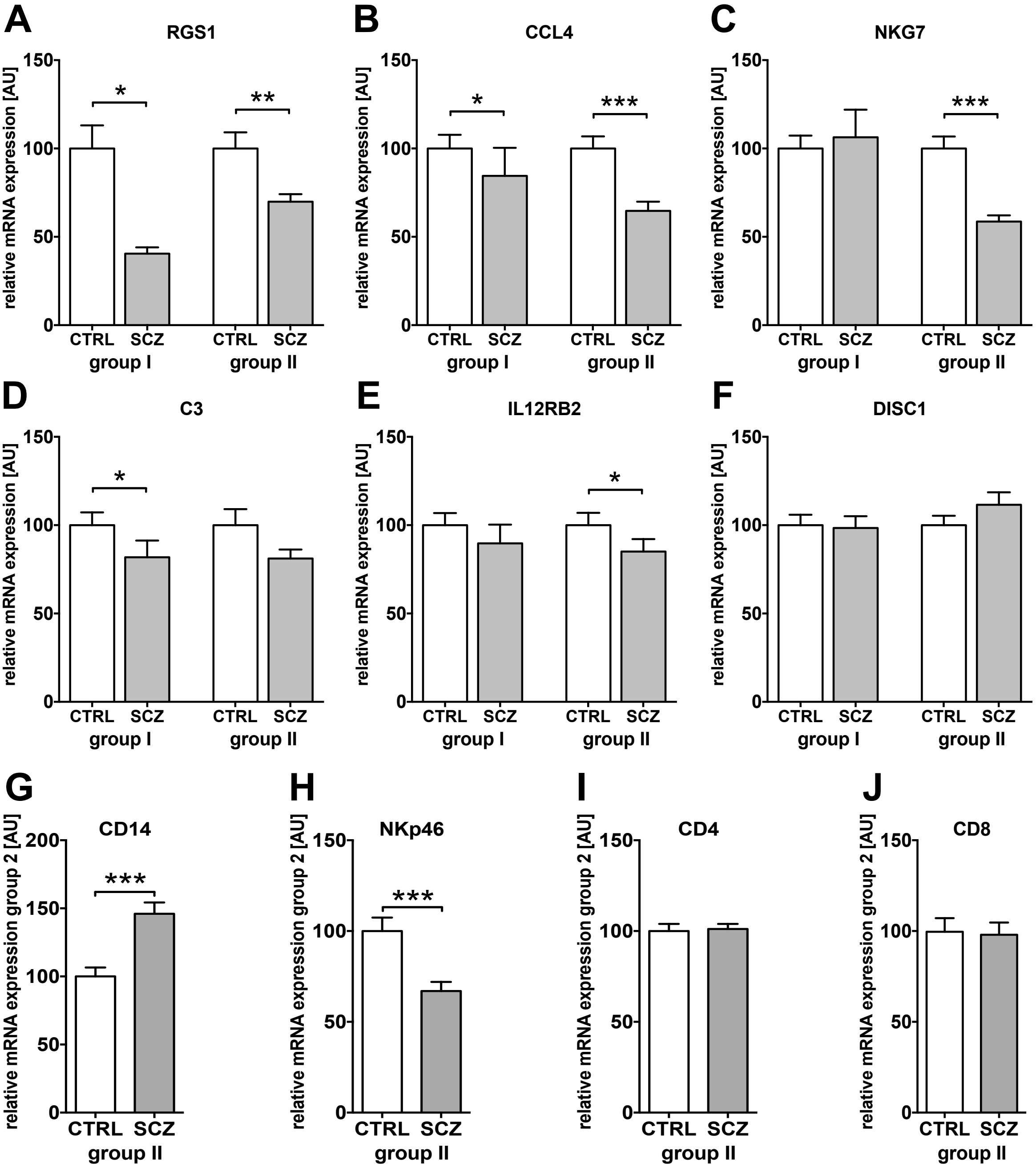

Supplementary Figure S5: Validation of microarray hits deregulated in PBMCs of tgDISC1 rats by quantitative Real-Time PCR in human PBMCs derived from two independent cohorts of patients diagnosed with schizophrenia (grey bars) and control subjects (light bars). Subjects of group I are depicted on the left side, subjects of independent group II are shown on the right side of each bar graph. (**A**) Reduced expression

of RGS1 was found in SCZ patients of both groups. Group I U-test *$P = 0.033$; group II U-test **$P = 0.006$. (**B**) Reduced mRNA levels of CCL4 were seen in SCZ subjects of both cohorts. Group I T-test *$P = 0.017$; group II T-test ***$P < 0.001$. (**C**) A reduction of expression levels of NKG7 could be shown in SCZ patients of group II. Group I U-test $P = 0.909$; T-test ***$P < 0.001$. (**D**) Group I showed a reduced expression of C3 in SCZ patients. Group I T-test *$P = 0.016$; Group II U-test $P = 0.337$. (**E**) Levels of IL12RB2 mRNA were decreased in SCZ subjects of group II. Group I U-test $P = 0.630$; Group II U-test *$P = 0.038$. (**F**) DISC1 mRNA levels were unchanged in both cohorts. Group I T-test $P = 0.505$; U-test $P = 0.564$. (**G**) Levels of CD14 mRNA, a marker for monocytes, were strongly increased in SCZ patients. Group II T-test ***$P < 0.001$. (**H**) In contrast, expression of NKp46, a marker for natural killer cells, was reduced in SCZ patients. Group II -test ***$P < 0.001$. (**I**) No change in CD4 mRNA, a marker for CD4-positive T-cells, could be detected in group II. T-test $P = 0.806$. (**J**) Equal expression of CD8B, a marker for $CD8^+$ T cells. Group II T-test $P = 0.866$. Abbreviations: CTRL, control subjects; SCZ, schizophrenic patients; AU, arbitrary units. All means ± s.e.m.

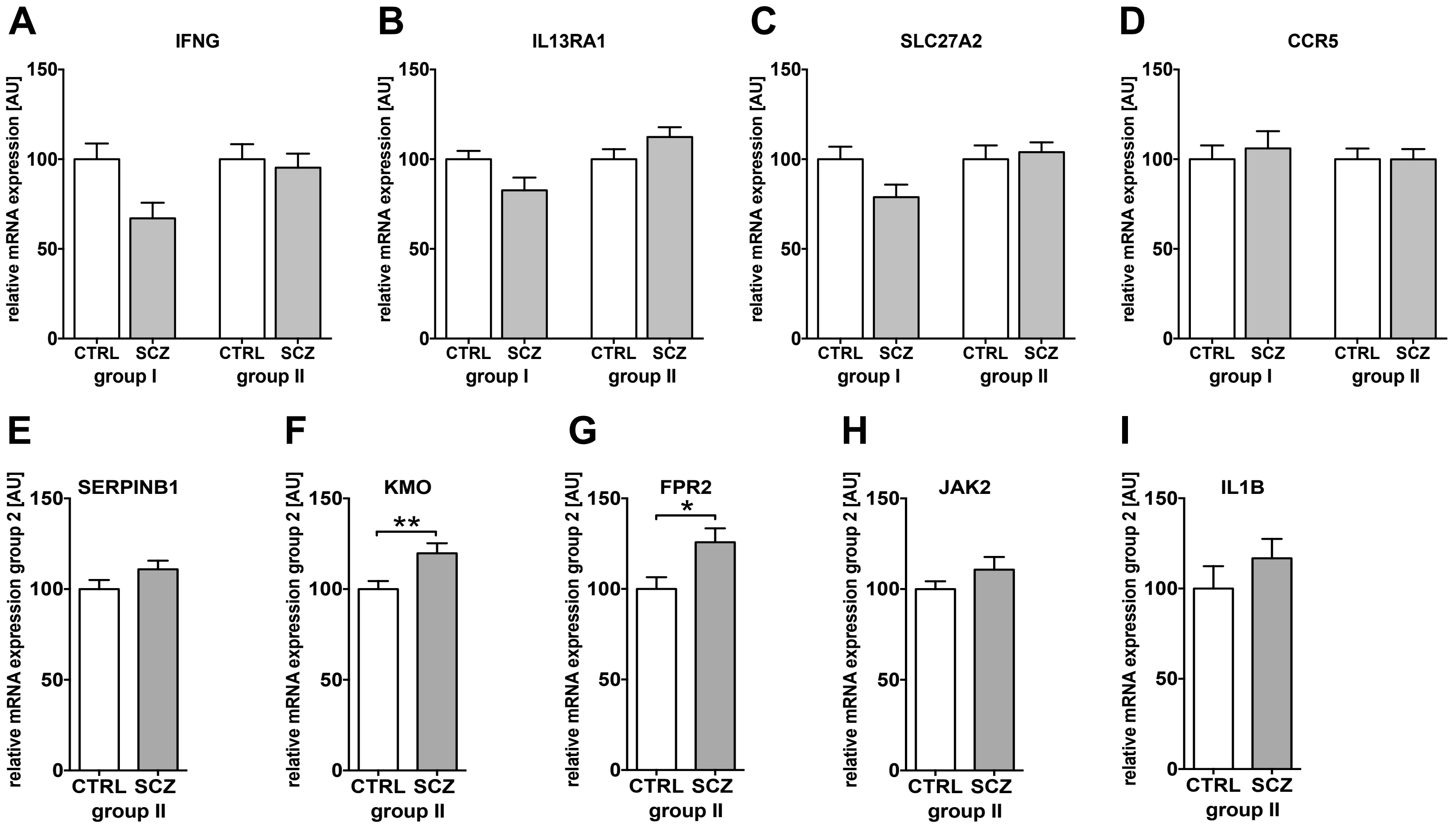
A
IFNG
B
IL13RA1
C
SLC27A2
D
CCR5
relative mRNA expression [AU]
150
100
50
0
CTRL
SCZ
group I
group II
E
SERPINB1
F
KMO
**
G
FPR2
*
H
JAK2
I
IL1B
relative mRNA expression group 2 [AU]

Supplementary Figure S6: Validation of further microarray hits deregulated in PBMCs of tgDISC1 rats by qPCR in human PBMCs derived from two independent cohorts of patients diagnosed with schizophrenia (grey bars) and control subjects (light bars). (**A**) No change in expression of IFNG was shown for both groups. Group I U-test *P* = 0.113; group II U-test *P* = 0.504. (**B**) Expression of IL13RA1 was not significantly changed. Group I T-test *P* = 0.077; group II U-test *P* = 0.098. (**C**) Expression of SLC27A2 was comparable between groups. Group I T-test *P* = 0.099; group II *U*-test *P* = 0.109. (**D**) No change in expression levels of CCR5 was seen in either group. Group I T-test *P* = 0.538; group II T-test *P* = 0.903. (**E**) No change in expression levels of SERPINB1 was detected in group II. U-test *P* = 0.114. (**F**) Group II SCZ patients exhibited increased expression levels of KMO. Group II T-test ***P* = 0.007. (**G**) Increased mRNA levels of FPR2 were measured in SCZ. Group II U-test **P* = 0.013. (**H**) No change in expression levels of JAK2 was detected. Group II U-test *P* = 0.437. (**I**) IL1B levels were comparable between CTRL and SCZ. Group II U-test *P* = 0.121. All means ± s.e.m.

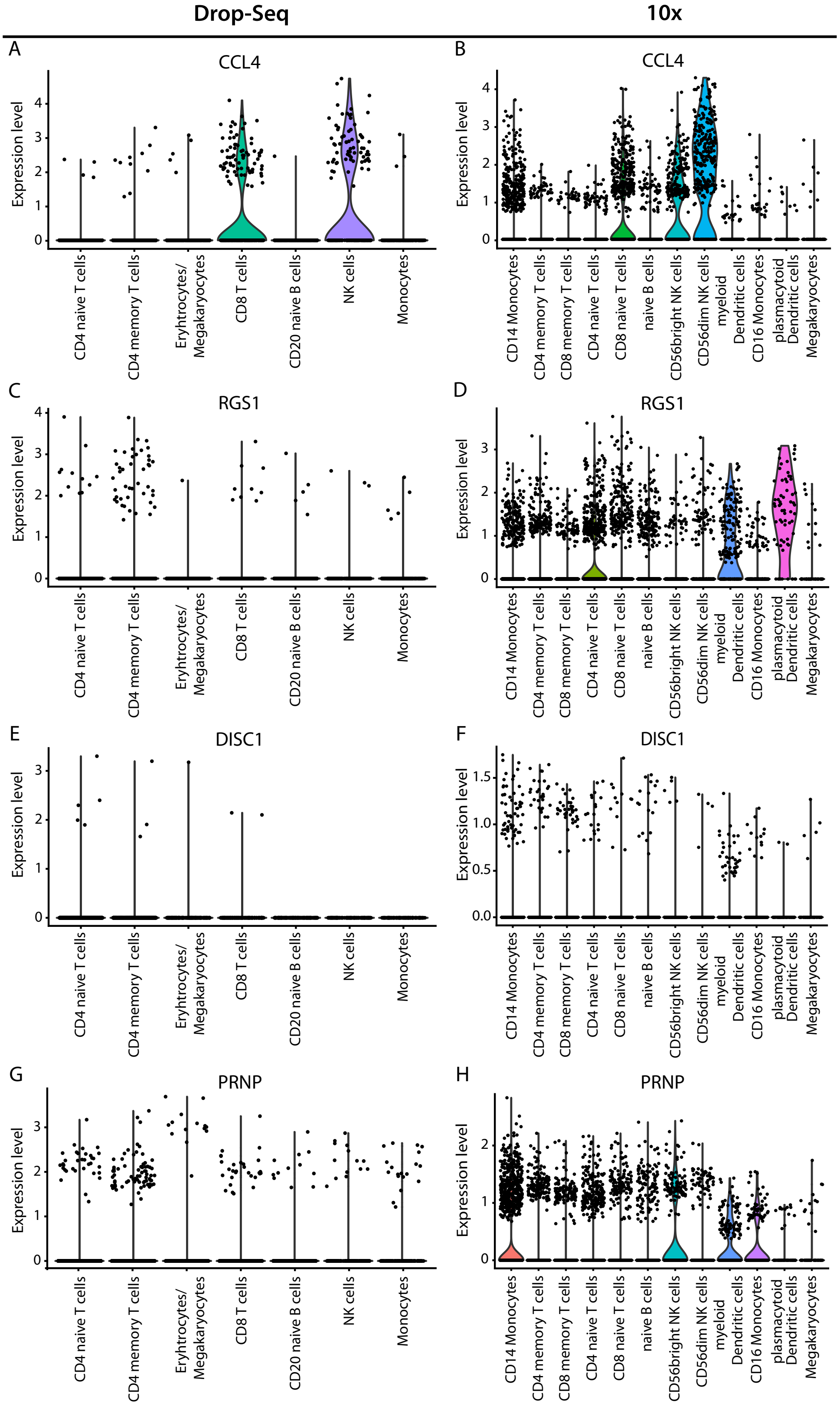
Drop-Seq
10x
A
CCL4
B
CCL4
C
RGS1
D
RGS1
E
DISC1
F
DISC1
G
PRNP
H
PRNP
Expression level
CD4 naive T cells
CD4 memory T cells
Eryhtrocytes/ Megakaryocytes
CD8 T cells
CD20 naive B cells
NK cells
Monocytes
CD14 Monocytes
CD8 memory T cells
CD8 naive T cells
naive B cells
CD56bright NK cells
CD56dim NK cells
myeloid Dendritic cells
CD16 Monocytes
plasmacytoid Dendritic cells
Megakaryocytes

Supplementary Figure S7: Expression profile of targets in human PBMC subclasses. Gene expression profile of selected marker genes from two independent single cell RNA-sequencing datasets (see Methods for description). (**A**), (**B**) it is unclear whether the observed decrease in CCL4 expression can be explained by the decrease in NK cell count, as CCL4 is also expressed in CD8-positive T cells. (**C**), (**D**) CD4 positive T cells and macrophages constitute the predominant source of RGS1. (**E**), (**F**) DISC1 is expressed at low levels in PBMCs, whereas the Prion protein (PRNP) is strongly expressed in all PBMC subtypes. Note that in the tgDISC1 rat the transgene is expressed under the hamster Prnp promoter (*1*). The data show that there is Prnp promoter-driven overexpression but not ectopic expression of DISC1 when human PBMCs are taken for comparison.

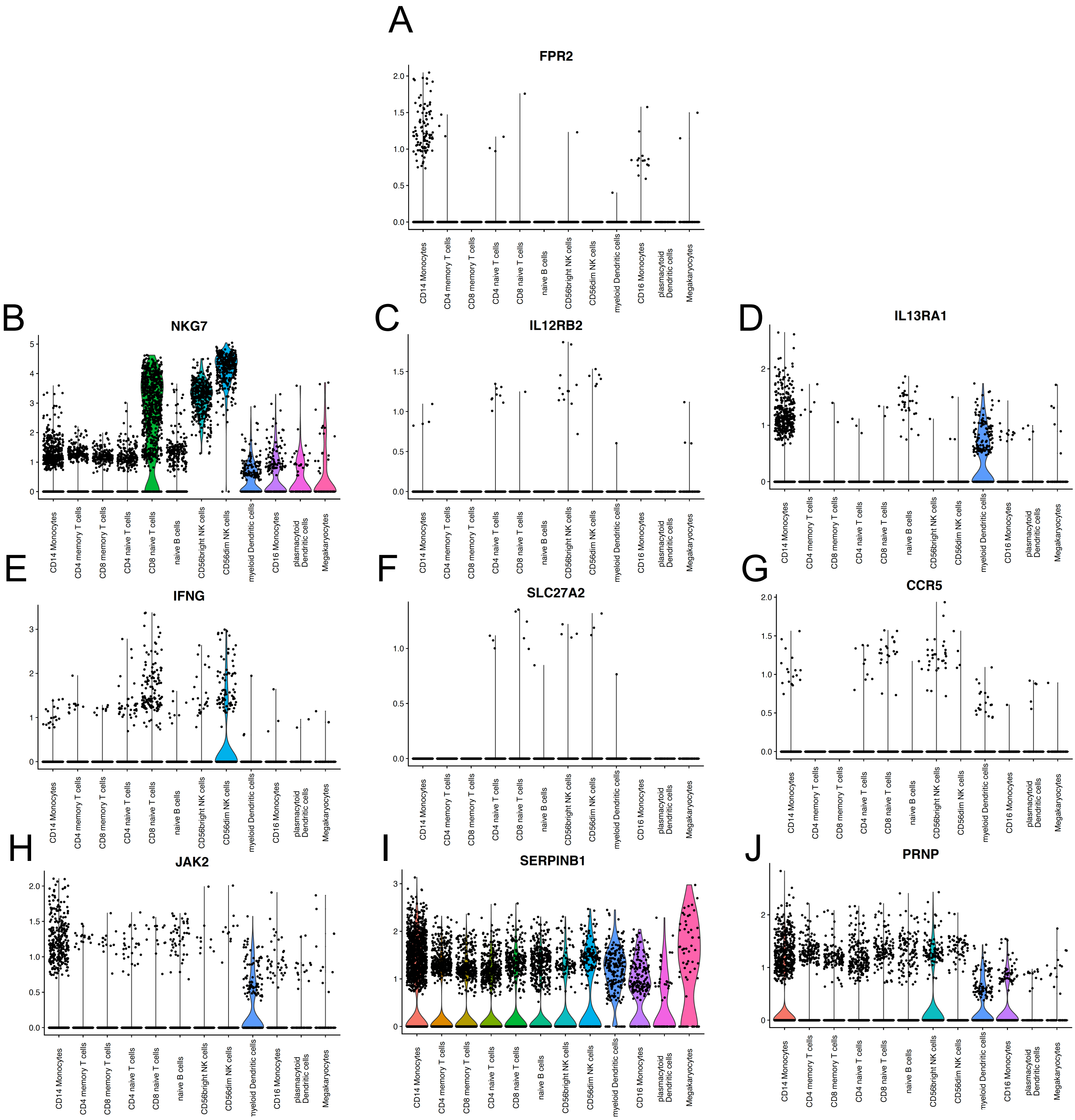

Supplementary Figure S8: Further expression data of transcripts in human PBMC subpopulations. Gene expression profile of selected marker genes from the larger RNA-sequencing dataset (*2*).

CTRL

		CCL4	NKG7	KMO	FPR2	IL12RB2	IFNG	IL13RA1	SLC27A2	SERPINB1	CCR5	DISC1	C3	JAK2	IL1B	CD14	NKp46	CD4	CD8
RGS1	r =	0.079	-0.025	.416**	0.148	0.051	0.095	0.081	.338*	.423**	.400*	0.031	0.192	0.138	0.138	-0.184	0.123	0.133	-0.056
	P =	0.632	0.881	0.006	0.344	0.754	0.553	0.608	0.031	0.008	0.014	0.844	0.254	0.414	0.445	0.291	0.496	0.428	0.737
	n =	39	38	42	43	40	41	42	41	38	37	42	37	37	33	35	33	38	38
CCL4	r =		.608**	-0.137	-.434**	0.2	.370*	-.310*	-0.018	0.188	-0.116	-.351*	0.239	-0.062	0.07	-.364*	.459**	-0.192	0.268
	P =		0,000	0.394	0.005	0.215	0.019	0.048	0.912	0.273	0.5	0.027	0.166	0.724	0.702	0.038	0.008	0.263	0.113
	n =		36	41	41	40	40	41	39	36	36	40	35	35	32	33	32	36	36
NKG7	r =			-0.258	-.502**	0.199	.370*	-.464**	0.187	-0.045	-0.009	-0.257	-0.011	-0.145	-0.194	-.393*	.419*	-0.145	.378*
	P =			0.113	0.001	0.238	0.022	0.003	0.247	0.799	0.962	0.114	0.951	0.42	0.313	0.026	0.019	0.413	0.028
	n =			39	40	37	38	39	40	34	33	39	33	33	29	32	31	34	34
KMO	r =				.447**	0.173	0.022	.303*	-0.004	0.198	0.306	0.284	0.192	.400*	0.204	0.116	-0.141	0.23	-0.179
	P =				0.002	0.279	0.891	0.048	0.981	0.228	0.062	0.065	0.249	0.013	0.248	0.501	0.426	0.159	0.276
	n =				44	41	42	43	42	39	38	43	38	38	34	36	34	39	39
FPR2	r =					-0.086	-0.159	.493**	-0.028	0.063	0.217	.344*	-0.048	.322*	.386*	.629**	-0.305	0.127	-0.258
	P =					0.59	0.309	0.001	0.857	0.704	0.192	0.022	0.775	0.048	0.024	0,000	0.079	0.441	0.112
	n =					42	43	44	43	39	38	44	38	38	34	36	34	39	39
IL12RB2	r =						0.109	-0.197	.403*	-0.026	0.201	.309*	-0.045	0.065	0.188	-0.158	0.057	-.414*	0.263
	P =						0.498	0.216	0.01	0.879	0.239	0.05	0.797	0.706	0.312	0.379	0.756	0.012	0.122
	n =						41	41	40	36	36	41	35	36	31	33	32	36	36
IFNG	r =							-0.209	.366*	0.061	.331*	0.028	0.279	0.19	-0.019	-0.178	0.296	-0.061	.501**
	P =							0.184	0.019	0.719	0.045	0.861	0.1	0.268	0.916	0.315	0.1	0.72	0.002
	n =							42	41	37	37	42	36	36	32	34	32	37	37
IL13RA1	r =								-0.212	0.197	0.054	0.127	0.153	0.295	0.246	.555**	-0.084	0.241	-.370*
	P =								0.178	0.236	0.752	0.418	0.367	0.076	0.161	0.001	0.641	0.146	0.022
	n =								42	38	37	43	37	37	34	35	33	38	38
SLC27A2	r =									0.165	.706**	0.241	-0.01	0.059	-0.158	-0.049	-0.104	0.052	.534**
	P =									0.329	0,000	0.124	0.956	0.733	0.388	0.78	0.564	0.758	0.001
	n =									37	36	42	36	36	32	35	33	37	37
SLC27A2	r =										0.272	0.064	-0.236	0.282	0.152	0.17	0.111	-0.116	-0.135
	P =										0.099	0.703	0.153	0.087	0.391	0.321	0.532	0.483	0.414
	n =										38	38	38	38	34	36	34	39	39
CCR5	r =											0.314	0.249	.443**	-0.047	0.055	-0.205	0.171	.346*
	P =											0.058	0.138	0.006	0.793	0.753	0.252	0.305	0.033
	n =											37	37	37	33	35	33	38	38
DISC1	r =												-0.042	.325*	0.274	.343*	-.401*	-0.072	0.146
	P =												0.807	0.05	0.123	0.044	0.021	0.668	0.382
	n =												37	37	33	35	33	38	38
C3	r =													0.268	0.171	-0.201	-0.008	0.287	0.132
	P =													0.109	0.332	0.247	0.965	0.08	0.431
	n =													37	34	35	33	38	38
JAK2	r =														.435*	0.277	-.365*	0.158	-0.131
	P =														0.011	0.107	0.037	0.343	0.433
	n =														33	35	33	38	38
IL1B	r =															.448*	0.083	0.02	-.400*
	P =															0.01	0.668	0.911	0.019
	n =															32	29	34	34
CD14	r =																-0.235	-0.073	-.384*
	P =																0.203	0.671	0.021
	n =																31	36	36
NKp46	r =																	-0.141	0.121
	P =																	0.427	0.494
	n =																	34	34
CD4	r =																		-0.189
	P =																		0.248
	n =																		39

SCZ

		CCL4	NKG7	KMO	FPR2	IL12RB2	IFNG	IL13RA1	SLC27A2	SERPINB1	CCR5	DISC1	C3	JAK2	IL1B	CD14	NKp46	CD4	CD8
	r =	-0.145	0.073	0.029	-0.093	0.012	-0.005	-0.212	.371**	0.046	0.224	0.136	0.124	0.214	0.09	-0.064	-0.144	-0.2	0.017
**RGS1**	*P* =	0.311	0.605	0.841	0.519	0.935	0.973	0.132	0.006	0.756	0.125	0.353	0.418	0.149	0.564	0.668	0.346	0.165	0.909
	n =	51	52	50	50	51	54	52	54	48	48	49	45	47	43	47	45	50	50
	r =		.682**	-0.169	-0.102	.475**	.421**	-0.258	-0.188	-0.049	0.015	-0.172	0.144	0.004	-0.037	-0.27	.819**	-0.179	0.108
**CCL4**	*P* =		0,000	0.256	0.494	0	0.002	0.074	0.185	0.75	0.922	0.253	0.358	0.978	0.817	0.077	0,000	0.228	0.47
	n =		49	47	47	50	51	49	51	45	45	46	43	44	41	44	43	47	47
	r =			-0.15	-0.145	0.23	.406**	-.280*	-0.101	-0.246	0.118	-0.089	0.281	-0.076	-0.266	-.463**	.480**	-0.224	0.126
**NKG7**	*P* =			0.31	0.319	0.112	0.003	0.049	0.475	0.099	0.434	0.552	0.065	0.619	0.092	0.001	0.001	0.126	0.392
	n =			48	49	49	52	50	52	46	46	47	44	45	41	45	43	48	48
	r =				0.211	0,000	0.112	-0.086	0.009	0.053	0.083	.566**	0.031	0.191	0.277	0.101	-0.158	-0.092	-0.098
**KMO**	*P* =				0.16	0.999	0.437	0.555	0.951	0.727	0.588	0,000	0.847	0.215	0.084	0.52	0.317	0.544	0.519
	n =				46	47	50	49	50	45	45	45	41	44	40	43	42	46	46
	r =					-0.187	-0.123	.336*	-0.03	0.045	-0.113	0.092	0.081	0.152	.411**	.693**	0.024	0,000	-.300*
**FPR2**	*P* =					0.209	0.393	0.019	0.839	0.767	0.466	0.549	0.606	0.325	0.009	0,000	0.88	0.999	0.043
	n =					47	50	48	50	45	44	45	43	44	39	43	41	46	46
	r =						.295*	-0.222	0.013	0.159	0.24	0.24	0.019	0.189	0.187	-0.125	.388*	-.319*	0.103
**IL12RB2**	*P* =						0.035	0.126	0.928	0.298	0.112	0.108	0.904	0.22	0.241	0.414	0.011	0.029	0.492
	n =						51	49	51	45	45	46	43	44	41	45	42	47	47
	r =							-0.245	0.169	-0.2	.322*	0.037	0.177	0.232	0.03	-0.23	0.29	0.058	0.103
**IFNG**	*P* =							0.08	0.222	0.173	0.026	0.801	0.245	0.116	0.847	0.12	0.053	0.687	0.478
	n =							52	54	48	48	49	45	47	43	47	45	50	50
	r =								-0.121	.395**	-0.018	-0.045	-0.028	0.14	0.069	.444**	-0.147	0.021	-.430**
**IL13RA1**	*P* =								0.394	0.006	0.907	0.766	0.861	0.354	0.669	0.002	0.348	0.888	0.002
	n =								52	47	46	47	43	46	41	45	43	48	48
	r =									0.043	.378**	.343*	0.128	0.259	0.273	-0.032	-0.252	-0.108	0.051
**SLC27A2**	*P* =									0.773	0.008	0.016	0.403	0.079	0.077	0.828	0.094	0.453	0.723
	n =									48	48	49	45	47	43	47	45	50	50
	r =										.434**	0.058	-0.246	0.014	-0.004	.412**	-0.012	-0.096	0.065
**SLC27A2**	*P* =										0.003	0.713	0.111	0.924	0.98	0.005	0.938	0.516	0.661
	n =										46	43	43	46	41	45	42	48	48
	r =											0.202	-0.025	0.032	0.117	-0.039	0.07	-0.029	-0.043
**CCR5**	*P* =											0.195	0.874	0.836	0.467	0.801	0.66	0.845	0.771
	n =											43	43	45	41	45	42	48	48
	r =												0.258	.683**	0.283	-0.021	-0.133	-0.139	0.061
**DISC1**	*P* =												0.109	0,000	0.081	0.894	0.406	0.361	0.689
	n =												40	42	39	43	41	45	45
	r =													0.189	0.074	0.093	0.141	-0.018	-0.157
**C3**	*P* =													0.224	0.656	0.557	0.393	0.907	0.303
	n =													43	39	42	39	45	45
	r =														0.224	0.174	-0.05	0.063	0.008
**JAK2**	*P* =														0.165	0.258	0.757	0.674	0.96
	n =														40	44	41	47	47
	r =															.478**	-0.011	-0.016	-0.201
**IL1B**	*P* =															0.002	0.947	0.917	0.196
	n =															40	37	43	43
	r =																-0.146	0.079	-0.167
**CD14**	*P* =																0.363	0.596	0.261
	n =																41	47	47
	r =																	-0.222	0.016
**NKp46**	*P* =																	0.148	0.916
	n =																	44	44
	r =																		-0.012
**CD4**	*P* =																		0.936
	n =																		50

Supplementary Figure S9: **Detailed correlation parameters between top hits expression levels measured in human subject samples of cohort II.** Detailed description of correlation shown in Figure 3A. All correlations have been performed using Spearman's ranked test.. Upper panel gives information for CTRL subjects, lower panel for SCZ subjects. Dark blue color indicates correlations that appear in both, SCZ and CTRL subjects; light blue color marks correlations seen only in CTRL subjects; correlations only appearing in SCZ patients are depicted in red color, color-coding for physiological (light to dark blue) to more pathological (red) relations. Depicted are the *P*-value, the correlation coefficient r and the *n* for every correlation.

References

1. S. V. Trossbach, V. Bader, L. Hecher, M. E. Pum, S. T. Masoud, I. Prikulis, S. Schable, M. A. de Souza Silva, P. Su, B. Boulat, C. Chwiesko, G. Poschmann, K. Stuhler, K. M. Lohr, K. A. Stout, A. Oskamp, S. F. Godsave, A. Muller-Schiffmann, T. Bilzer, H. Steiner, P. J. Peters, A. Bauer, M. Sauvage, A. J. Ramsey, G. W. Miller, F. Liu, P. Seeman, N. J. Brandon, J. P. Huston, C. Korth, Misassembly of full-length Disrupted-in-Schizophrenia 1 protein is linked to altered dopamine homeostasis and behavioral deficits. *Mol. Psychiatry* **21**, 1561-1572 (2016).

2. G. X. Zheng, J. M. Terry, P. Belgrader, P. Ryvkin, Z. W. Bent, R. Wilson, S. B. Ziraldo, T. D. Wheeler, G. P. McDermott, J. Zhu, M. T. Gregory, J. Shuga, L. Montesclaros, J. G. Underwood, D. A. Masquelier, S. Y. Nishimura, M. Schnall-Levin, P. W. Wyatt, C. M. Hindson, R. Bharadwaj, A. Wong, K. D. Ness, L. W. Beppu, H. J. Deeg, C. McFarland, K. R. Loeb, W. J. Valente, N. G. Ericson, E. A. Stevens, J. P. Radich, T. S. Mikkelsen, B. J. Hindson, J. H. Bielas, Massively parallel digital transcriptional profiling of single cells. *Nat. Commu.n* **8**, 14049 (2017).
